# Hypoxia-induced lncRNA-AC020978 promotes proliferation and glycolytic metabolism of non-small cell lung cancer by regulating PKM2/HIF-1α axis

**DOI:** 10.7150/thno.43839

**Published:** 2020-03-26

**Authors:** Qian Hua, Baoming Mi, Fei Xu, Jun Wen, Li Zhao, Jianjun Liu, Gang Huang

**Affiliations:** 1 Department of Nuclear Medicine, Renji Hospital, School of Medicine, Shanghai Jiaotong University, Shanghai 200127, China.; 2 Shanghai Key Laboratory of Molecular Imaging, Shanghai University of Medicine and Health Sciences, Shanghai 201318, China.; 3 Department of Nuclear Medicine, The second Affiliated Hospital of Soochow University, Suzhou, Jiangsu 215004, China.

**Keywords:** Long noncoding RNAs, AC020978, PKM2, HIF-1α, aerobic glycolysis

## Abstract

**Rationale**: Non-small cell lung cancer (NSCLC) is a deadly disease with a hallmark of aberrant metabolism. The mechanism of glycolysis associated lncRNA underlying the aggressive behaviors of NSCLC is poorly understood.

**Methods**: The expression level of AC020978 in NSCLC was measured by quantitative real-time PCR and fluorescence *in situ* hybridization (FISH) assay. The biological role of AC020978 in cell proliferation and aerobic glycolysis was determined by functional experiments *in vitro* and *in vivo*. The transcription of AC020978 was assessed by dual-luciferase reporter and chromatin immunoprecipitation (ChIP) assay. RNA pull-down, mass spectrometry and RNA immunoprecipitation (RIP) assays were used to identify the interaction protein with AC020978. Western blotting, *in situ* proximity ligation assay (PLA), and co-immunoprecipitation (co-IP) were performed to reveal the potential mechanism of AC020978.

**Results**: The present study indicated that AC020978 was upregulated in NSCLC, significantly correlated with advanced TNM stage and poor clinical outcomes, representing as an independent prognostic predictor. Functional assays revealed AC020978's role in promoting cell growth and metabolic reprogramming. Moreover, AC020978 was an upregulated lncRNA under glucose starvation as well as hypoxia conditions, and directly transactivated by HIF-1α. Mechanistic investigations identified that AC020978 directly interacted with Pyruvate kinase isozymes M2 (PKM2) and enhanced PKM2 protein stability. Besides, this study uncovered that AC020978 could promote the nuclear translocation of PKM2 and regulate PKM2-enhanced HIF-1α transcription activity.

**Conclusions**: Together, these data provided evidence that AC020978 conferred an aggressive phenotype to NSCLC and was a poor prognosticator. Targeting AC020978 might be an effective therapeutic strategy for NSCLC.

## Introduction

Lung cancer is the most common cancer and the leading cause of cancer-related death worldwide according to the latest annual global cancer statistics report 1. Non-small cell lung cancer (NSCLC) accounts for approximately 83% of primary lung cancer and nearly 80% of NSCLC patients are diagnosed with advanced stage due to lack of early detection biomarkers 2. Patients with advanced lung cancer are exposed to a poor prognosis. Thus, identifying potential biomarkers and developing therapeutic targets is in urgent need for early diagnosis and effective intervention of this disease.

During the rapid growth of solid tumors, delayed tumor angiogenesis leads to a shortage of glucose supply and an oxygen deprivation microenvironment to cancer cells 3,4. To survive, cancer cells have evolved complicated regulatory networks to adapt to these harsh conditions. One common feature of tumor to harness cellular stress is the alteration of energy metabolism, known as aerobic glycolysis or Warburg effect, which has been pointed out as an emerging hallmark of cancer recent years 5. This metabolic shift relies on glycolysis rather than mitochondrial oxidative phosphorylation (OXPHOS) to generate energy, which has been correlated with a series of pathological mechanisms 4,6. Another characteristic of tumor gained widespread attention is the activation of hypoxia-inducible factor-1 (HIF-1) pathway, a contributor to many aggressive biological behavior of cancer cells and poor clinical outcome of patients 3. HIF-1 participates in promoting metabolic reprogramming by transactivating multiple hypoxia-responsive genes related to glycolytic metabolism, vice versa, Warburg effect assists cancer cells to adjust their microenvironment to thrive, reciprocally promoting the malignant feature of NSCLC 7-9.

The pathogenesis of NSCLC involves multiple complicated genetic and epigenetic alterations 2. Recently, long noncoding RNAs (lncRNAs) have been identified as a new regulator in the progression of cancers with diverse functions and mechanisms 10. LncRNAs are transcripts longer than 200 nucleotides in length with little-no protein coding capacity 10. To date, emerging evidence has suggested that cancer-associated lncRNA plays an important role in the establishment of metabolic rewiring 11-13. Analysis of the correlation between metabolic reprogramming and aberrant lncRNA expression may highlight a novel insight into the metabolic aspects in initiation, promotion and progression of NSCLC.

Since the fact that hundreds of protein-coding genes are transactivated by HIF-1 under hypoxic conditions, whether lncRNAs could be responsible for hypoxia and their regulatory roles is far from clear 14. Mole et al. 15 undertook the integrated genomic analyses of both non-coding and coding transcripts in hypoxic cells. Their analysis implicated that non-coding RNA was also hypoxia-inducible. Since then, dysregulated lncRNA expression targeted by HIF-1 in several cancer types has been described 16,17. However, there are still many unknown lncRNAs which may potentially be the molecular response to hypoxia and their functional mechanism in modulating cellular adaption need to be further elucidated.

In our previous study, RNA-seq has been applied to identify glycolysis associated lncRNAs between NSCLC tissues with high ^18^F-fluorodeoxyglucose (FDG) uptake and their adjacent normal lung tissues 18. LncRNA-AC020978 is among the top-scoring upregulated lncRNAs in the RNA-seq expression profiling data. Clinical analysis reveals that upregulation of AC020978 is correlated with poor prognosis of NSCLC patients. Functional assay demonstrates its oncogenic role in promoting proliferation as well as glycolysis pathway. Besides, AC020978 is inducible under glucose starvation as well as hypoxia conditions, and directly transactivated by HIF-1α. Mechanistically, we find that AC020978 directly binds with PKM2, which is a key rate-limiting enzyme to catalyze the conversion of phosphoenolpyruvate (PEP) and ADP to pyruvate acid and generates ATP at the last step of glycolysis. Aberrant expression of PKM2 is a most frequent pathogenic subtype in cancers 19. Here, we observe that AC020978 stabilizes PKM2 protein from ubiquitination degradation and promotes PKM2 nuclear translocation, as well as facilitates PKM2-enhanced HIF-1α transcriptional activity. AC020978/PKM2/HIF-1α positive feedback loop triggers a cascade reaction in aerobic glycolytic and cancer progression, which may be a promising metabolism blocker target for antitumor therapy.

## Materials and Methods

### Patients and clinical samples

NSCLC specimens and adjacent tissues were acquired from the surgical specimen archives of Renji Hospital, School of Medicine, Shanghai Jiaotong University. This research was approved by the institutional clinical research ethics committee of Renji Hospital, School of Medicine, Shanghai Jiaotong University. Written informed consent was obtained from each patient and the study was conducted in accordance with the International Ethical Guidelines for Biomedical Research Involving Human Subjects (CIOMS). None of these patients had received radiotherapy or chemotherapy prior to surgery. All patients were staged based on the criteria of the 8th Edition of the Lung Cancer Staging Manual 20.

### Cell culture and treatment

Human NSCLC cell lines, A549, H1299, H1650, H1975, PC9, human normal lung epithelial cell line HBE and human diploid fibroblast IMR-90 cells were purchased from ATCC. All cell lines were tested for mycoplasma contamination before used to ensure that they were mycoplasma-free.

The small interfering RNAs (siRNAs) against human AC020978, PKM2, and HIF-1α were transfected using Lipofectamine 2000 (Invitrogen) according to the manufacturer's instruction, while non-specific siRNA was used as negative control. All the siRNAs were purchased from Genepharma Technology. AC020978 vector (0978-OE) and PKM2 vector (PKM2-OE) were subcloned into the expression vector pcDNA-4/TO (Invitrogen). The plasmids were transfected using the Neofect transfection reagent (Neofect biotech), while empty vector plasmid was used as negative control. Sh-0978 and sh-NC lenti-virus were purchased from GenePharma and constructed into A549 cell lines for further *in vivo* experiments. The sequences of the siRNA and sh-RNA were listed in Table S1.

### Total RNA extraction and quantitative real-time PCR

Total RNA was isolated by TRIzol Kit (Omega) and the quantity of total RNA was measured by NanoDrop equipment (Thermo Fisher Scientific). Total RNA was reverse transcribed using the cDNA Synthesis kit (Takara). Real-time PCR was performed using SYBR Green PCR Master Mix (Takara) in a StepOnePlus RT-PCR system (Thermo Fisher Scientific). The amplified transcript level of each specific gene was normalized to β-actin by using 2^-ΔΔCt^ method. The sequences of primers used in this study are listed in Table S1.

### Subcellular fractionation analysis

Subcellular isolation of RNA was performed with a PARIS™ Kit (Ambion) according to the manufacturer's instructions. The cytoplasmic and nuclear RNA were further analyzed by qRT-PCR. β-actin and U6 were used as cytoplasmic and nuclear controls, respectively. Protein fractionation of nuclear and cytosolic extracts was performed by using Minute^TM^ Nuclear and Cytoplasmic Extraction kit (Invent Biotechnologies) according to the manufacturer's instructions. Alpha Tubulin and lamin B1 were used as cytoplasmic and nuclear controls, respectively.

### Fluorescence *in situ* hybridization (FISH)

FISH analysis was performed according to a previously described method 21. Specific target probe sequence of AC020978 was listed in Table S1. AC020978 expression was quantified using a visual grading system based on the intensity of staining (0 = no staining, 1 = weak staining, 2 = moderate staining, 3 = strong staining) and the extent of staining (1 = 0-25%, 2 = 26-50%, 3 = 51-75%, 4 = 76-100%). The final scores were computed by multiplying the intensity score and the percentage score of positive cells. Samples were classified into two categories, high expression group (score 7-12) and low expression group (score 0-6).

### Cell proliferation

Cell proliferation was measured by the Cell Counting Kit-8 (bimake) according to the manufacturer's protocol. Briefly, 5 × 10^3^ control or treated cells were seeded onto 96-well plates per well. 10 μL CCK-8 solution was added at specified time points. After incubating at 37°C for 2 h, optical density at 450 nm (OD450) was measured for each sample. Cell proliferation was also assessed by the Ethynyldeoxyuridine (EdU) analysis (RiboBio) according to the manufacturer's instruction as reported previously 18.

### Glucose uptake and lactate production

^18^F-FDG uptake assay was performed as previously reported to reflect the intracellular glucose uptake level of cells 18. For lactate production measurements, cell supernatant was collected to measure lactate concentration (Nanjing Jiancheng Bioengineering Institute) according to the manufacturer's instructions. Results were normalized to total cell number of each sample.

### Extracellular Acidification Rate (ECAR) and Oxygen Consumption Rate (OCR) Assays

The Seahorse XF 24 Extracellular Flux Analyzer (Seahorse Bioscience) was used to monitor *in vitro* cells metabolic alternations. Cells, transfected with control siRNA, si-0978, control plasmid, and AC020978 overexpressing plasmid, were seeded in a XF 24-well plate at a density of 1 × 10^4^ per well and allowed to attach overnight, followed by serum starvation for 24 h. OCR was measured by Seahorse XF Cell Mito Stress Test kit (Agilent Technologies) and ECAR was detected using Seahorse XF Glycolysis Stress Test kit (Agilent Technologies) according to a previously described method 21. ECAR and OCR measurements were normalized to cell number and reported as mpH/min or pmoles/min, respectively.

### Western blot analysis

Western blot analysis has been done using standard technique 21. All the antibodies used in this study were listed in Table S2.

### Chromatin immunoprecipitation (ChIP) assay

ChIP was performed with a ChIP assay kit (Beyotime) according to the manufacturer's protocol. Briefly, A549 cells were cross-linked with formaldehyde and sonicated to an average size of 200-to-500 basepairs. Cell lysates were precleared with protein A/G beads before incubated with protein A/G beads coated with the anti-HIF-1α antibody (proteintech). Anti-rabbit IgG was used as a negative control. Cross-linked DNA released from the protein-DNA complex was purified by DNA Extraction Kit (GeneMark) and the eluted DNA was further subjected to qRT-PCR. The specific primers used for ChIP-qPCR are presented in Table S1.

### Luciferase reporter assay

AC020978 promoter region containing two HIF-1α putative binding area (wild type, WT) or mutant area (mutant type, MUT) was constructed into pGL3-based vectors. To determine the effect of HIF-1α on AC020978 promoter, pGL3-based construct containing AC020978 WT or MUT promoter sequences plus renilla luciferase reporter plasmid was individually transfected into HEK293 cells. Cultured under normoxia or hypoxia 24 h after transfection, firefly and renilla luciferase activity were measured by a dual-luciferase reporter assay system (Promega). The ratio of firefly luciferase to renilla activity was calculated for each sample.

### Biotin-RNA pull-down assay

The biotin-RNA pull-down assay was performed as described previously 22. Briefly, the full length AC020978 sequence was PCR amplified using a T7-containing primer and then reversely transcribed by MAXIscript™ T7 Transcription Kit (Thermo Fisher Scientific). The targeted RNA was biotin-labeled with Pierce™ RNA 3' End Desthiobiotinylation Kit (Thermo Fisher Scientific). A549 and H1299 cells were collected and lysed by protein lysis buffer, while Streptavidin magnetic beads were used to capture the biotin-labeled AC020978 probe. The biotinylated nucleic acid compounds were incubated with purified proteins using Pierce™ Magnetic RNA-Protein Pull-Down Kit (Thermo Fisher Scientific). The pull-down complexes were analyzed by mass spectrometry or western blot technique.

### RNA immunoprecipitation (RIP) assay

RIP was performed using a magna RNA-binding protein immunoprecipitation kit (Millipore) according to the manufacturer's instructions. The cell lysates were incubated with RIP buffer containing magnetic bead conjugated with human anti-PKM2 antibody (proteintech) or anti-mouse IgG. The co-precipitated RNAs were detected by qRT-PCR. To demonstrate that the detected RNA signals specifically bind to PKM2, total RNA (input controls) and IgG controls were simultaneously assayed.

### Co-immunoprecipitation (Co-IP) assay

Reciprocal endogenous co-IP assay was performed as reported previously 23. Information of used antibodies was described in Table S2.

### Proximity Ligation Assay (PLA)

Proximity Ligation Assay was performed using the Duolink® *In situ* Red Starter Kit Mouse/ Rabbit (Sigma-Aldrich). Briefly, A549 cells were grown on glass coverslips and fixed using 4% paraformaldehyde. The cells were washed with PBS containing glycine and permeabilized using 0.1% Triton X-100 in PBS for 20 min. After blocking, the cells were incubated with anti-PKM2 and anti- HIF-1α antibodies (proteintech) diluted in blocking solution overnight at 4°C. The subsequent step was incubating the pre-diluted anti-rabbit plus and anti-mouse minus probes at 37°C for 1h. Then, cells were incubated with 1× ligase for 30 min and 1× polymerase for 100 min at 37°C. Finally, coverslips were mounted on the slide with Duolink® *In situ* Mounting Medium with DAPI.

### Immunofluorescence (IF)

Immunofluorescence staining was performed as previously described 23. Expression and localization of the proteins were observed under a confocal microscope system (Olympus BX61). Information for antibodies used in immunofluorescence staining was described in Table S2.

### Xenograft mouse model and ^18^F-FDG micro-PET/CT

All experimental procedures were approved by the Institutional Animal Care and Use Committee of Renji Hospital, School of Medicine, Shanghai Jiao Tong University. In order to clarify the effect of AC020978 *in vivo*, 4-week male BALB/c nude mice obtained from Renji Hospital Experimental Animal Center were used in our study. The mice were inoculated subcutaneously with 1 × 10^7^ A549 cells in the right flank with sh-0978, while sh-NC in the left flank to establish the NSCLC xenograft model. Tumor size was measured every 3 days.

3 weeks after subcutaneous inoculation, micro-PET/CT scan (Super Nova® PET/CT, Pingseng Healthcare Inc.) was performed to measure ^18^F-FDG uptakes of xenograft tumor in the mice. ^18^F-FDG (0.1 mL, 7.4 MBq) was injected into the tail vein of tumor-bearing mice. After 30 min, the animals were anesthetized with 2% isoflurane and immobilized during 15 min PET scan acquisition. ^18^F-FDG uptake was assessed by the maximum standard uptake value (SUVmax) through drawing a region of interest.

### Immunohistochemistry (IHC)

IHC was performed and measured as reported previously 21.

### Statistical procedures

Statistical analysis was performed using SPSS 22.0 software and GraphPad Prism 8.0. The results are presented as mean ± SD. Data were examined whether normally distributed with the One-Sample Kolmogorov-Smirnov test. If they were normally distributed and the variation between two groups were comparable, the comparisons were performed using Student's t-test. The comparisons among three or more groups were firstly performed by one-way analysis of variance (ANOVA) test if the variation between groups were comparable. If the results showed significant difference, the Student Newman Keuls analysis was used to test the difference. For the clinicopathologic analysis, the Chi-square test or Fisher exact test (two-sided) were performed. The survival curves were calculated using the Kaplan-Meier method, and differences were assessed by a log-rank test. Cox multivariate regression analysis was used to determine the independent factors that influenced survival and recurrence. All results were reproduced across triplicate experiments.

## Results

### AC020978 is clinically relevant in NSCLC

AC020978 is among the top-scoring upregulated non-coding RNAs in the RNA-seq expression profiling data in our previous study 18. It is a 428 bp transcript with 2 exons and localizes in human chromosome 16, intron overlapping with the PRMT7 gene (Figure S1A). In order to validate the non-coding property of AC020978, we employed the sequence analysis program by ORF Finder from the National Center for Biotechnology Information and the result showed that it failed to predict a protein of more than 39 amino acids. The coding probability of AC020978 was as low as 0.029 if any, as calculated by Coding-Potential Assessment Tool (CPAT). In addition, codon substitution frequency (CSF) analysis indicated that AC020978 did not have protein-coding potential (Figure S1B). The full-length sequence of AC020978 was obtained by rapid amplification of the 5' and 3' cDNA ends (RACE) assays. Sequencing of PCR products revealed the boundary between the universal anchor primer and AC020978 (Figure S1C).

To confirm the expression pattern of AC020978, qRT-PCR assay was performed on 16 paired NSCLC tissues and the corresponding adjacent normal tissues. Results showed that AC020978 expression was much higher in tumor tissues than adjacent tissues (Figure S2A). Additionally, the expression level of AC020978 was relatively high in five NSCLC cell lines compared to normal lung cells IMR-90 and HBE (Figure S2B).

**Figure 1:**
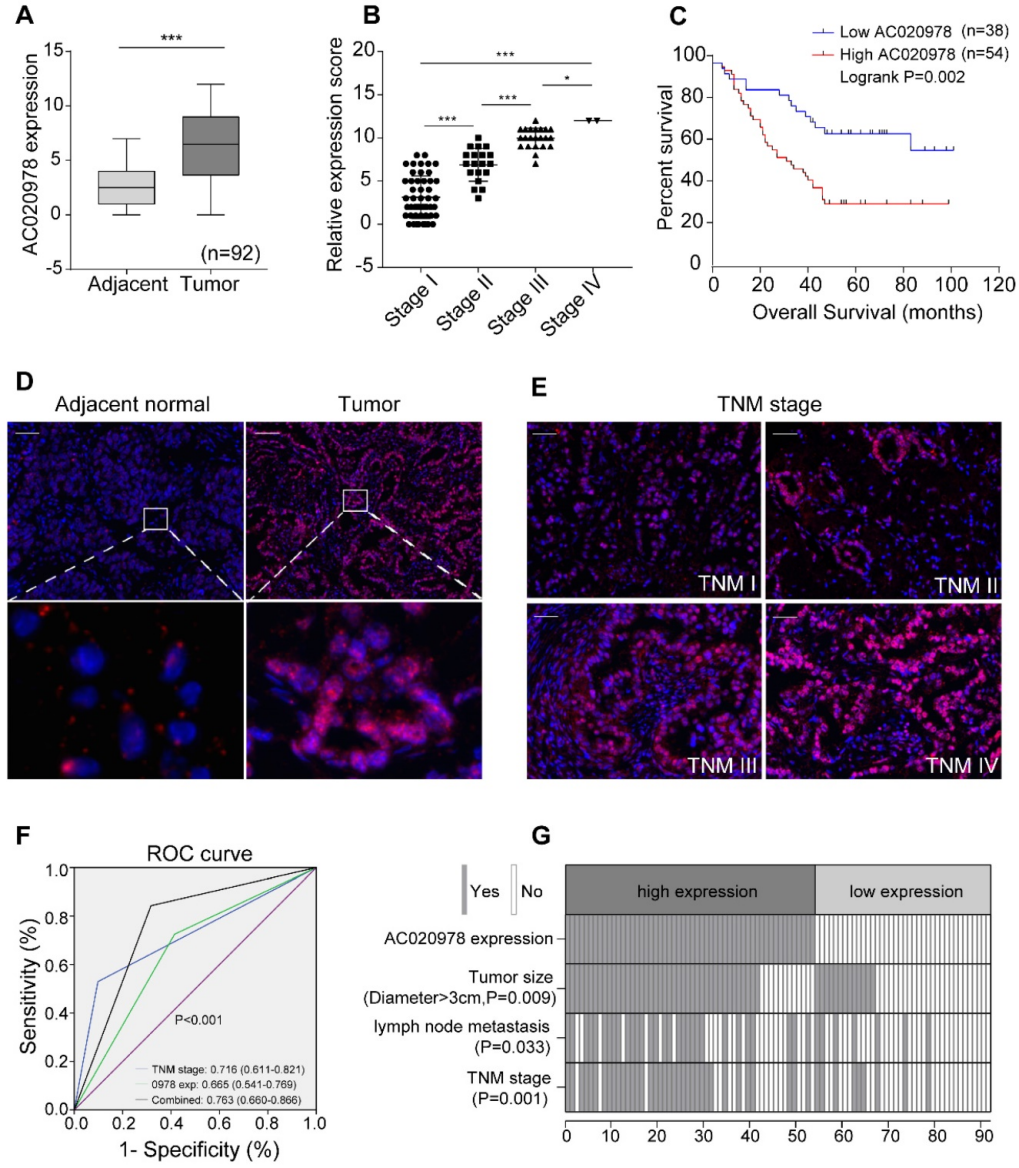
** LncRNA candidate AC020978 is clinically relevant in NSCLC. (A)** Statistical analysis of AC020978's FISH expression score in 92 pairs of NSCLC tissues and adjacent normal tissues, paired t-test. In boxplots (middle line depicts the median and the whiskers the min to max. range). **(B)** Relative AC020978's FISH expression levels in different TNM stages. **(C)** Survival is analyzed and compared between patients with low and high levels of AC020978 in 92 NSCLC patients, log-rank test.( high expression: score 7-12; low expression: score 0-6). **(D-E)** Representative FISH images of AC020978 expression in NSCLC tumor tissues and in different TNM stages (blue, DAPI; red spot, positive staining; Scale bar = 100 µm). **(F)** ROC analysis of AC020978-based, TNM-based and the combination model in predicting clinical outcome. **(G)** The heatmap illustrates the association of different clinical characters with AC020978 high and low-expression tumors. Data shown are mean±SD (n = 3). (*P < 0.05, **P < 0.01, ***P < 0.001).

To investigate the clinical significance of AC020978 in cancer, we performed FISH assay in an additional 92 paraffin-embedded NSCLC and adjacent tissues. The 92 paired samples were stratified into high expression (score 7-12) and low expression group (score 0-6) based on AC020978 expression score. As shown in Figure 1A and 1D, AC020978 was overexpressed in tumor tissues and rarely expressed in the adjacent non-tumor tissues. Besides, the expression of AC020978 was increased with advanced TNM stage (Figure 1B, 1E). Kaplan-Meier analysis revealed that high expression of AC020978 was significantly associated with a poor prognosis in these patients (P < 0.01, Figure 1C).

To further evaluate the pathological and clinical predictive value of AC020978, receiver operating characteristic curve (ROC) analysis was performed among AC020978-based, TNM-based and the combination of AC020978 and TNM-based group to predict clinical outcomes. Data of area under curve (AUC) indicated that the combination model (0.763) was higher than TNM-based model alone (0.716) (Figure 1F), suggesting that the combination of AC020978 and TNM stage was more precise in predicting clinical outcome than TNM stage alone. Subsequently, we evaluated and compared AC020978 expression with different clinicopathological features and found that AC020978 expression was significantly associated with tumor size (P < 0.01), lymph node metastasis (P < 0.05) and TNM stage (P < 0.01) (Figure 1G, Table S3). Univariate and multivariate regression analyses demonstrated that AC020978 expression was an independent prognostic indicator for NSCLC patients with significant hazard ratios [HR, 2.274; 95% confidence interval (CI), 1.184-4.366; P=0.014; Table S4]. Collectively, these results suggested that AC020978 might be a potential biomarker in NSCLC.

### AC020978 is an oncogenic lncRNA in NSCLC cells

**Figure 2:**
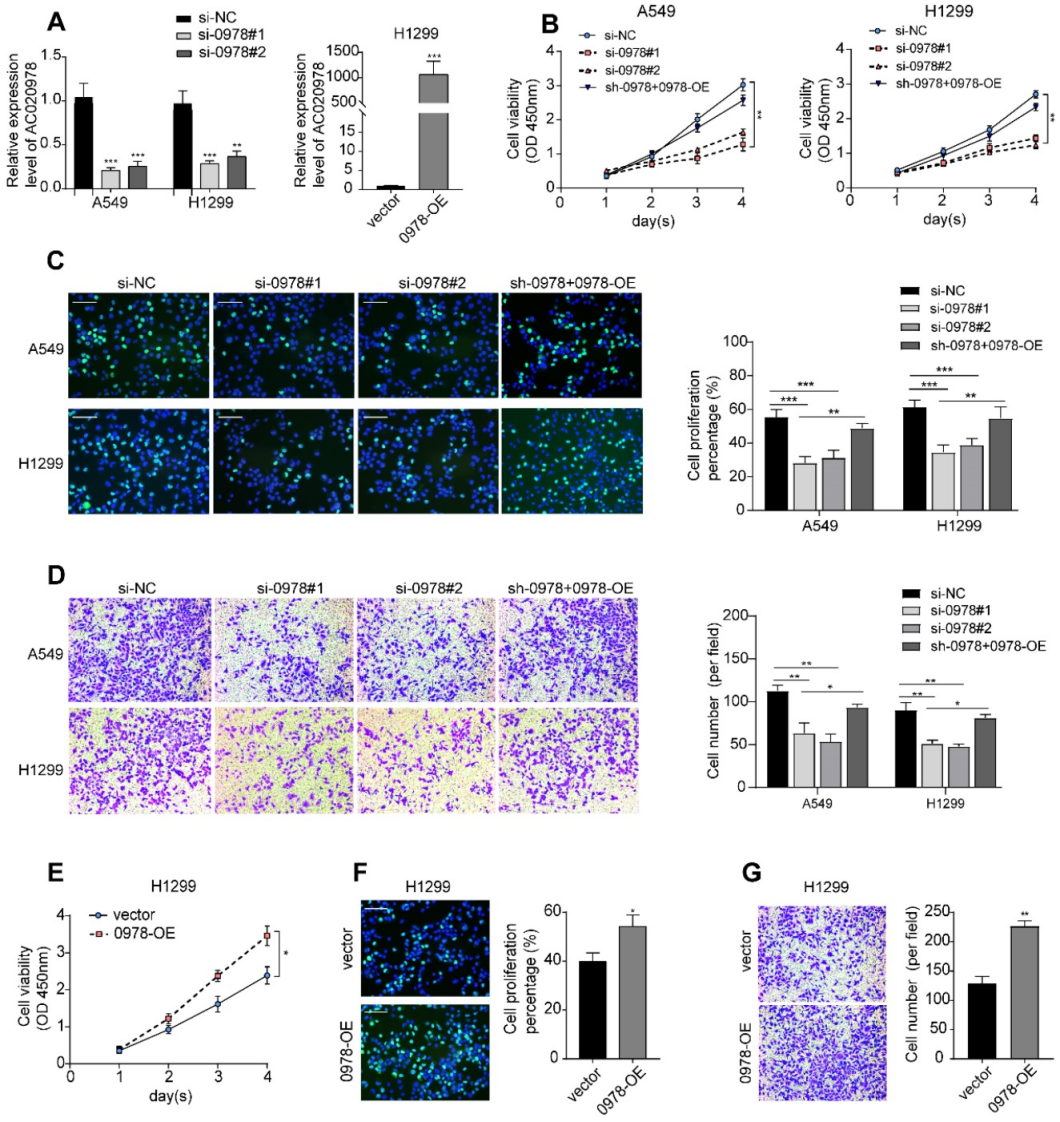
** AC020978 is an oncogenic lncRNA in NSCLC cells. (A)** The inhibiting efficiency of two different AC020978 siRNAs in both A549 and H1299 cells. Relative expression levels of AC020978 in H1299 cell transfected with pcDNA-0978 plasmid. **(B)** Cell proliferation was performed by CCK-8 assay over a 4-day period in A549 and H1299 cells transfected with negative control group (si-NC), AC020978 siRNAs group (si-0978#1, si-0978#2) and rescue group (sh-0978+0978-OE). **(C)** Immunofluorescence analysis of Edu was performed in in A549 and H1299 cells transfected with negative control group (si-NC), AC020978 siRNAs group (si-0978#1, si-0978#2) and rescue group (sh-0978+0978-OE) after 24 h. **(D)** Transwell assay was performed in in A549 and H1299 cells transfected with negative control group (si-NC), AC020978 siRNAs group (si-0978#1, si-0978#2) and rescue group (sh-0978+0978-OE) after 24 h. **(E)** Cell proliferation was performed by CCK-8 assay over a 4-day period in H1299 cells transfected with pcDNA-0978 or vector control. **(F)** Immunofluorescence analysis of Edu was performed in AC020978 overexpression cells after 24 h. **(G)** Transwell assay was performed in AC020978 overexpression cells after 24 h. Scale bar = 100 µm. Data shown are mean±SD (n = 3). (*P < 0.05, **P < 0.01, ***P < 0.001).

To elucidate the function of AC020978 on cell biological behavior, we transfected A549 and H1299 cells with two different siRNAs and each siRNA could effectively knock down AC020978 expression. Si-0978#1 showed the strongest suppression of AC020978 and was used throughout this study. The whole length of AC020978 overexpression vector was constructed and transfected into H1299 cell. Following qRT-PCR verified that the expression of AC020978 could increase over 1,000 fold (Figure 2A).

To investigate the biological roles of AC020978 in NSCLC progression, cell proliferation was analyzed using CCK-8, EdU staining, and colony formation assay. All these results showed that cell proliferative capability was remarkably attenuated following knockdown of AC020978 in A549 and H1299 cells. In addition, subsequent re-expression of AC020978 in stable AC020978-knockdown cells could rescue the attenuated cell proliferation rate (Figure 2B-C, Figure S2C). In the gain-of-function assays, overexpression of AC020978 accelerated the proliferative effects in H1299 cell (Figure 2E-2F, Figure S2D).

Transwell assay was utilized to measure the migratory ability of AC020978 in NSCLC cells. The results demonstrated that the ability of migration was increased in H1299 cell with ectopic expression of AC020978, while down-regulation of AC020978 suppressed the migratory activities of A549 and H1299 cells compared with the negative control, and re-expression of AC020978 in stable AC020978-knockdown cells could rescue the attenuated cell migratory activities (Figure 2D and 2G). It can be concluded that AC020978 is effective in facilitating NSCLC cell growth and migration.

### AC020978 is induced under metabolic stress and is pivotal to glycolytic metabolism reprogramming

**Figure 3:**
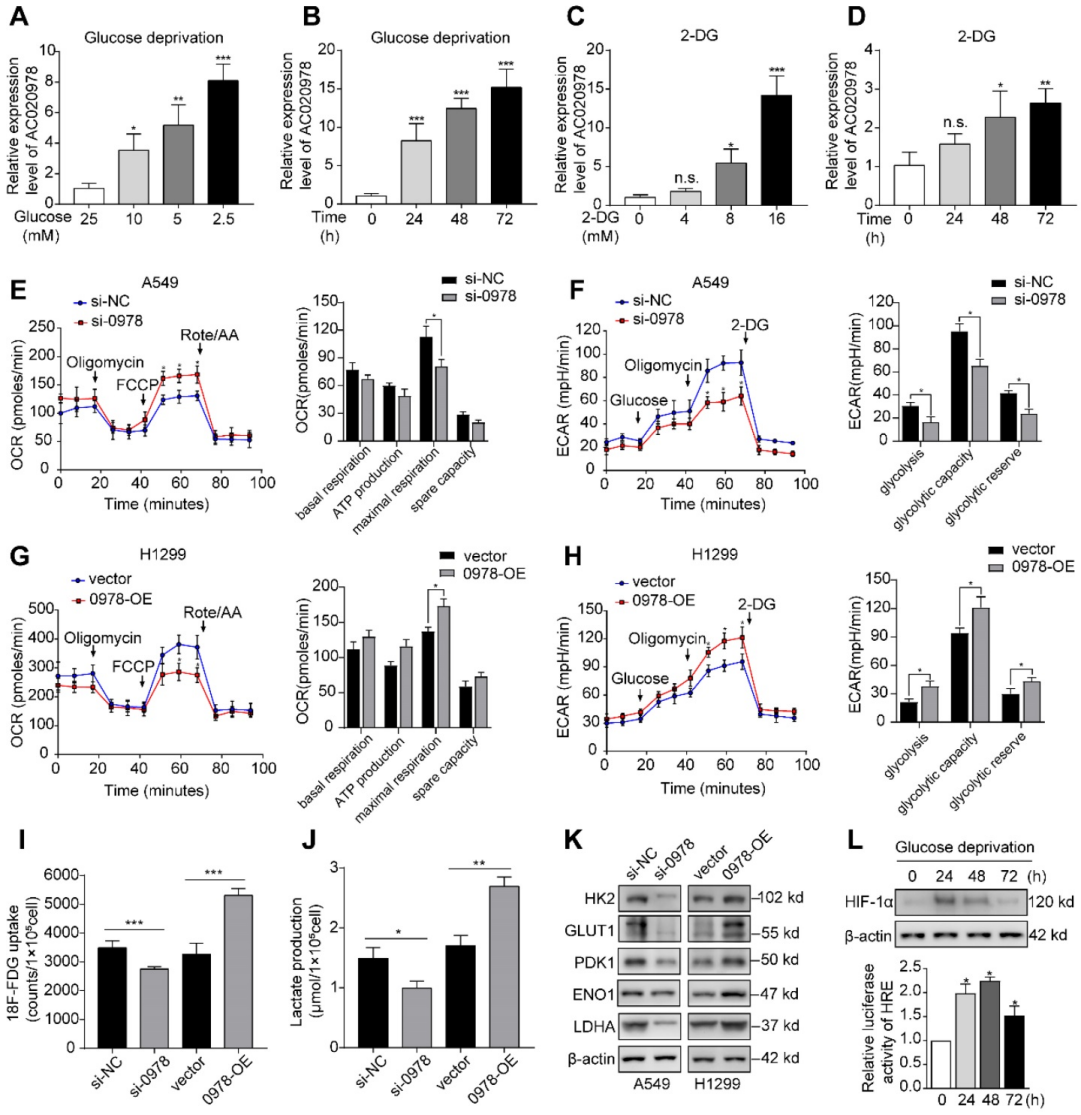
** AC020978 is induced under metabolic stress and enhances glycolytic metabolism. (A-B)** QPCR results showed that AC020978 was upregulated under low glucose culture conditions compared to normal glucose in dose-dependent manner (A, culture for 24 h) and time-dependent manner (B, glucose 2.5 mM). **(C-D)** QPCR results showed that AC020978 was increased after 2-DG treatment in dose-dependent manner (C, culture for 72h) and in time-dependent manner (D, 2-DG 5 mM). **(E)** Oxygen Consumption Rate (OCR) upon cells were measured by Seahorse XF after transfecting with si-NC or si-0978 in A549 cells. The OCR curves treated with oligomycin, FCCP and rotenone/antimycin A. Black arrows indicate the time point of cell treatment. **(F)** Extracellular acid ratio (ECAR) upon cells were measured by Seahorse XF after transfecting with si-NC or si-0978 in A549 cells. The ECAR curves treated with glucose, oligomycin and 2-DG. Black arrows indicate the time point of cell treatment. **(G)** The change of OCR level with different treatment in H1299 cells after transfecting with empty vector or pcDNA-0978. **(H)** The change of ECAR level with different treatment in H1299 cells after transfecting with empty vector or pcDNA-0978. **(I)** Relative^ 18^F-FDG uptake level was determined in A549 cells transfected with si-NC or si-0978 and in H1299 cells transfected with control or AC020978 overexpression plasmid. **(J)**Relative lactate release level was determined in A549 cells transfected with si-NC or si-0978 and in H1299 cells transfected with control or AC020978 overexpression plasmid. **(K)** HK2, GLUT1, PDK1, ENO1 and LDHA protein levels were examined under AC020978 knockdown and overexpression conditions by western blotting. **(L)** HIF-1α protein expression and HIF-1α responsive luciferase reporter were examined under low glucose culture conditions (glucose 2.5 mM) at the indicated time point. Data shown are mean±SD (n = 3). (*P < 0.05, **P < 0.01, ***P < 0.001).

It is known that glucose deprivation is a common characteristic of solid tumors. To elucidate whether AC020978 played a role in cellular survival under metabolic stress, we used varying glucose concentration gradient from 2.5 mM to 25 mM to mimic glucose deprivation conditions. Moreover, the glycolysis inhibitor 2-deoxyglucose (2-DG) was utilized to block glucose supply. As shown in Figure 3A-3D, AC020978 was induced by either glucose deprivation or 2-DG treatment in a dose-dependent and time-dependent manner, indicating that AC020978 might hold a critical role in cell adaptation to metabolic stress.

Next, whether AC020978 directly influenced glucose metabolism was measured by Seahorse XF Cell Mito Stress and Glycolysis Stress Test, which are the standard assay to reflect metabolic flux distribution of glucose. As shown in Figure 3E and 3F, silencing of AC020978 increased the maximal respiration in OCR assay, while ECAR assay showed that AC020978 knockdown markedly reduced glycolysis, glycolytic capacity and glycolytic reserve in A549 cells. On the contrast, overexpression of AC020978 showed an opposite metabolic flux in H1299 cell (Figure 3G and 3H), suggesting that AC020978 facilitated glycolysis pathway. In addition, a significant reduction of ^18^F-FDG uptake and lactate production was detected in A549 cell with AC020978-downregulation, while ectopic induction of AC020978 in H1299 cell showed reversal effect (Figure 3I-J). Furthermore, we measured the protein levels of several glucose transporter and metabolic enzymes. Western blotting results showed that alteration of AC020978 expression could positively affect the glucose transporter-1 (GLUT1), hexokinase II (HK2), pyruvate dehydrogenase kinase 1 (PDK1), enolase 1 (ENO1) and lactate dehydrogenase A (LDHA) protein level (Figure 3K). These collecting data demonstrated that AC020978 was a glucose starvation-induced lncRNA and could regulate glycolytic metabolism in NSCLC.

Since HIF-1α is a transcription factor involved in the regulation of genes important for cellular adaption to hypoxia and low glucose supply, the protein levels and transactivation of HIF-1α was analyzed under glucose starvation condition (Figure 3L). The results showed that both HIF-1α expression and HIF-1α responsive luciferase reporter could be induced by low glucose (2.5 mM) incubation. Intriguingly, compared to longer incubation period, 24 h had the highest HIF-1α protein expression, while 48 h had the highest HIF-1α responsive luciferase activity, indicating that cancer cells might constantly be adapted to their metabolic stress microenvironment.

### AC020978 promotes tumor growth and aerobic glycolysis *in vivo*

**Figure 4:**
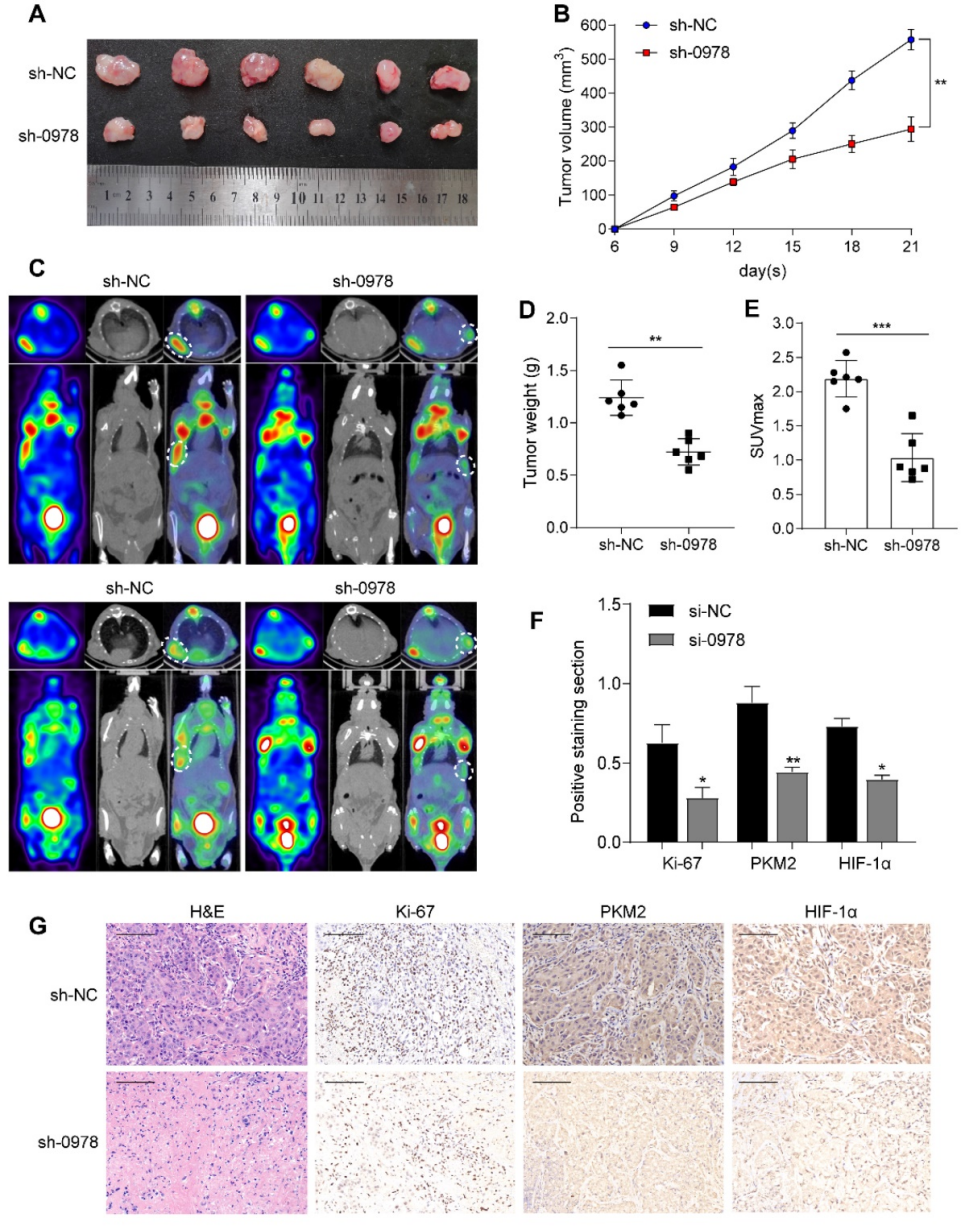
** AC020978 promotes tumorigenesis and aerobic glycolysis *in vivo*. (A)** Image of xenograft tumors resected from nude mice-bearing A549 cells with or without AC020978 knockdown (n=6). **(B)** Tumor growth curves showed that sh-0978 group suppressed tumor growth compared with sh-NC group. **(C)** Representative ^18^F-FDG micro-PET/CT images of two living nude mice were conducted 3 weeks after subcutaneous inoculation. Images showed obvious FDG uptake in sh-NC xenografts, while mild FDG uptake in sh-0978 xenografts. **(D)** Tumor weight was measured in mice after different treatments. **(E)** Comparison of the maximum standard ^18^F-FDG uptake value (SUVmax) of tumor tissues between sh-NC and sh-0978 group. **(F)** Quantification of the proliferation index (Ki-67 proportion), positive staining sections of PKM2 and HIF-1α levels in the tumor sections between sh-NC and sh-0978 group. **(G)** Representative images of H&E staining and IHC staining of Ki-67, PKM2 and HIF-1α from the tumor sections between sh-NC and sh-0978 group. Scale bar = 100 µm (200x). Data shown are mean±SD (n = 3). (*P < 0.05, **P < 0.01, ***P < 0.001).

To explore the roles of AC020978 *in vivo*, we established stable A549 cell lines with AC020978 knockdown and transplanted into nude mice subcutaneously to generate xenograft models. As shown in Figure 4A-B and 4D, knockdown of AC020978 dramatically reduced tumor volume and tumor weight compared to control group. Additionally, *in vivo* glycolytic metabolism was supported by ^18^F-FDG micro-PET/CT imaging. Results showed that xenografts derived from control group had a relative strong accumulation of ^18^F-FDG (mean SUVmax: 2.35), while ^18^F-FDG uptake in AC020978 downregulation group was much lower (mean SUVmax: 1.08; Figure 4C, 4E). IHC staining with ki-67, PKM2 and HIF-1α of xenograft tissues was in favor of AC020978's role in mediating tumor growth and glycolysis (Figure 4F-4G). Collectively, *in vivo* results were consistent with *in vitro* data, jointly proved that AC020978 played a crucial role in promoting proliferation and glycolysis of NSCLC.

### AC020978 is hypoxia-inducible and directly transactivated by HIF-1α

**Figure 5:**
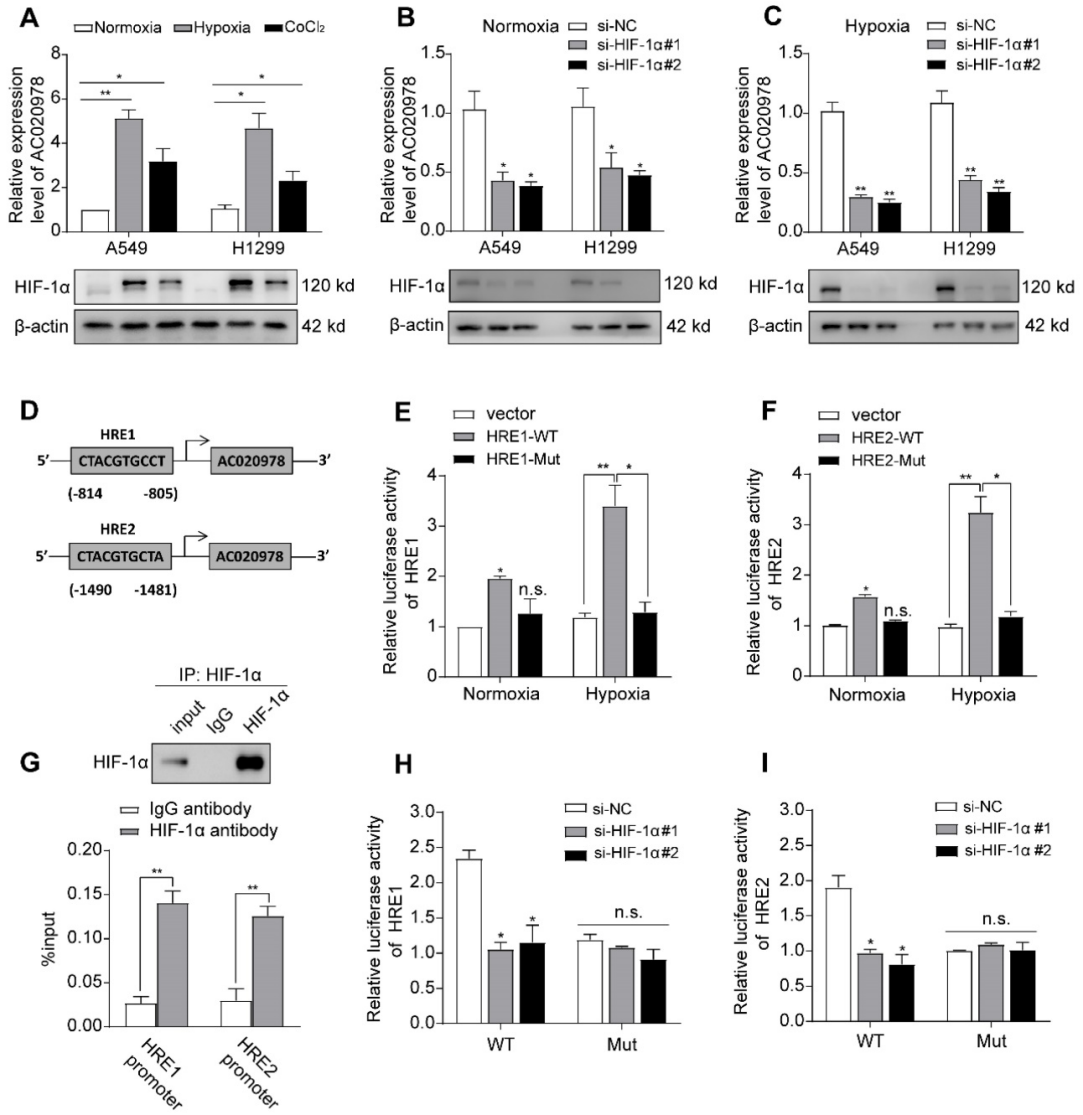
** AC020978 is a direct transcriptional target of HIF-1α. (A)** The expression levels of AC020978 (upper) and HIF-1α protein (lower) in A549 and H1299 cells were measured after culturing under normoxia, hypoxia (1% O2) or CoCl_2_ (100 μM) for 24 h by qRT-PCR and Western blot. **(B)** The expression of AC020978 was evaluated by qRT-PCR in A549 and H1299 cells after knockdown of HIF-1α with two siRNAs (si-HIF-1α#1, si-HIF-1α#2) under normoxia condition. **(C)** The expression of AC020978 was evaluated by qRT-PCR in A549 and H1299 cells after knockdown of HIF-1α with two siRNAs (si-HIF-1α#1, si-HIF-1α#2) under hypoxia condition. **(D)** Schematic illustration of two putative HIF-1α binding sites (HRE1 and HRE2) in AC020978 gene promoter. **(E-F)** HEK293 cells were transfected with pGL3 reporter vector containing AC020978 wide-type promoter (HRE-WT), or mutant-type promoter (HRE-MUT), respectively. Those transfected cells were further treated under normoxia or hypoxia. After 48 h, firefly luciferase activity was detected and normalized by renilla activity. **(G)** ChIP assay with anti-HIF-1α antibody was performed to verify the binding between HIF-1α and two HREs in AC020978 promoter in A549 cells. **(H-I)** HEK293 cells were transfected with pGL3 reporter vector containing AC020978 wide-type promoter (HRE-WT), or mutant-type promoter (HRE-MUT), respectively. Those transfected cells were further treated with si-NC or siRNAs of HIF-1α (si-HIF-1α#1, si-HIF-1α#2). After 48 h, firefly luciferase activity was detected and normalized by renilla activity. Data shown are mean±SD (n = 3). (*P < 0.05, **P < 0.01, ***P < 0.001).

We next explored which factors induced high AC020978 expression in NSCLC. The genomic sequence upstream region (~2kb upstream) of the gene coding for AC020978 was inspected using the promoter sequence analysis tools (UCSC and JASPAR). Two putative HIF-1α binding sites were found within the promoter region (Figure 5D). After treatment with hypoxia or its chemical inducer CoCl_2_ for 24 h, the expression of AC020978 in A549 and H1299 cells was significantly elevated in accordance with upregulated HIF-1α expression (Figure 5A). On the contrary, knockdown HIF-1α dramatically inhibited AC020978 expression during both normoxia and hypoxia condition (Figure 5B-5C). The subsequent ChIP-qPCR assay supported the notion that HIF-1α directly binding to the chromatin fragments of the two predicted promoter regions of AC020978 gene (Figure 5G). To further validate the activation of HIF-1α on AC020978 transcription, DNA fragments containing wild-type or mutant HRE binding sequence of AC020978 were inserted into the promoter region of a firefly luciferase reporter plasmid. As was expected, hypoxia significantly enhanced the luciferase density in cells containing wild-type promoter (WT), but not in cells with mutant-type promoter (MUT). Nevertheless, knockdown of HIF-1α remarkably repressed the luciferase density containing WT promoter (Figure 5E-F, H-5I). Taken together, our data intensively indicated that AC020978 was a direct transcriptional target of HIF-1α.

### AC020978 physically binds with PKM2 and mediates the stability of PKM2 protein

**Figure 6:**
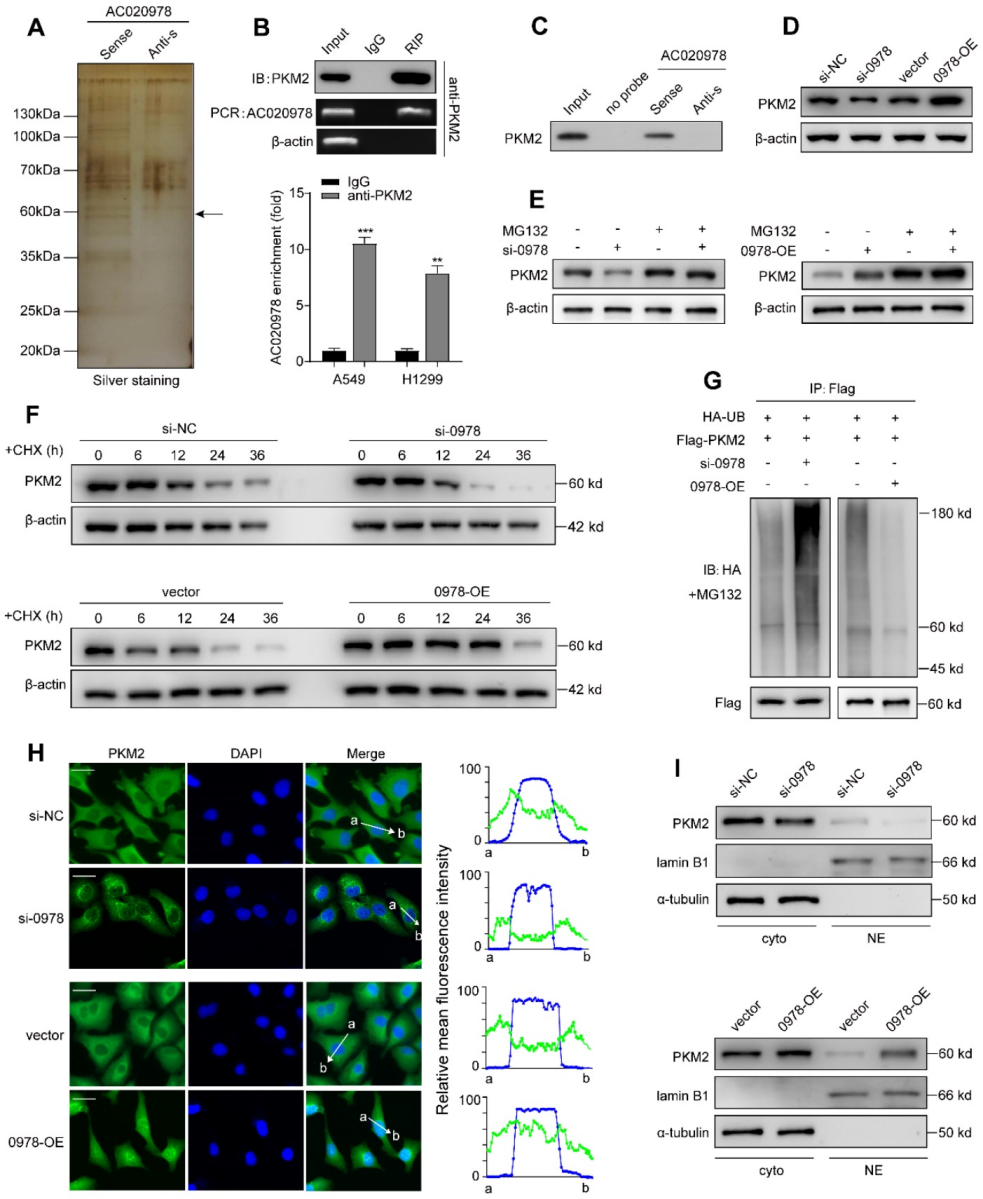
** AC020978 interacts with PKM2 and regulate the stability of PKM2 protein. (A)** Proteins retrieved from the AC020978 pull-down assay were analyzed by mass spectrometric analysis. **(B)** RIP assays using anti-PKM2 antibody showed that PKM2 interacted with AC020978 in A549 cells. The results of agarose electrophoresis of the PCR products are shown in the upper panel. The q-PCR results of RIP assays are shown in the bottom panel. **(C)** Western blot analysis of the proteins retrieved from the AC020978 pull-down assay, its antisense sequence was used as negative control. **(D)** Western blot showed that PKM2 protein level was positively regulated by AC020978 in A549 and H1299 cells. **(E)** A549 cell expressing either si-NC or si-0978 and H1299 cell expressing either vector or 0978-OE were treated with or without MG132 (50 µM) for 6 h. Cell lysates were analyzed by western blot with indicated antibodies. **(F)** A549 cell transfecting with si-NC or si-0978 and H1299 cell transfecting with pcDNA-vector or pcDNA-0978 were treated with cycloheximide (CHX, 100 ng/ml) for the indicated periods of time. Cell lysates were analyzed by western blot to examine PKM2 protein half-life. Protein band intensity was analyzed by ImageJ. **(G)** *In vitro* ubiquitination assay of cells transfected with si-0978 in A549 cell (left) or pcDNA-0978 in H1299 cell (right). All cells were co-transfected with flag-PKM2 and HA-UB plasmid, 42 h after transfection, cells were incubated with MG132 (50 µM) for 6 h. Cell lysates were immunoprecipitated with anti-flag antibody followed by immunoblotting analysis with anti-HA or anti-flag antibody. **(H)** Representative images of subcellular localization of PKM2 in A549 cells with different treatment by immunofluorescence. Cells were immunostained with anti-PKM2 (PKM2, green). The nucleus is marked with DAPI (blue). Merged images are shown. The line profiles of the mean fluorescence intensity of PKM2 and DAPI signals were measured by ImageJ software. (Scale bars=20 µm.) **(I)** Nuclear and cytosolic lysates were extracted from A549 and H1299 cells exposed to knockdown or overexpression of AC020978, followed by western blot analysis with indicated antibodies. Data shown are mean±SD (n = 3). (*P < 0.05, **P < 0.01, ***P < 0.001).

Increasing evidence has revealed that cytoplasm lncRNAs may behave as decoys for miRNAs or proteins 24. Since AC020978 mainly accumulated at cytoplasm (Figure S3A-S3B), RNA pull-down followed by mass spectrometry assay was conducted to screen AC020978-interacting proteins. As shown in Figure 6A, from the MS data files (Table S5), we paid particular attention to a RNA-binding candidate with confidence score > 100, PKM2, which was a key speed-limited enzyme in glycolysis, for further binding validation. Result of RIP assay indicated a robust and specific enrichment of AC020978 co-precipitated within PKM2 immunocomplex (Figure 6B). Western blot analysis of proteins retrieved from AC020978 pull-down assay confirmed that PKM2 protein specifically binds to the sense sequence of AC020978, but not to the antisense control (Figure 6C).

To interrogate which specific region within AC020978 contributes to PKM2 binding, we constructed four different deletion fragments of AC020978 based on the predicted secondary structure in the AnnoLnc database (http://annolnc.cbi.pku.edu.cn). Subsequently, RNA pull-down assay followed by western blotting showed PKM2 specific binding sequence was located within 248-428 nt long region. To further investigate which domain of PKM2 accounted for its interaction with AC020978, RIP assay was performed using a series of Flag-tagged PKM2 deletion mutants. The A2 domain (219-390) of PKM2 was essential for the binding of PKM2 to AC020978 (Figure S4A-S4B).

Next, we tried to explore the molecular function of this AC020978-PKM2 interaction. We found that alteration of AC020978 expression substantially regulated PKM2 protein level (Figure 6D). Upregulation of PKM2 protein in tumor tissues could be achieved by promoting transcription, inhibiting proteasome degradation or enhancing mRNA translation 19. However, there was no significantly difference of PKM2 mRNA level in AC020978 knockdown or overexpression cells (Figure S4C-S4D). Thus, we speculated that AC020978 might regulate the protein stability of PKM2. Notably, we observed that the reduced protein level of PKM2 by AC020978 downregulation was apparently recovered by the addition of MG-132 in A549 cells (Figure 6E). Results from cycloheximide (CHX) chase assay demonstrated that PKM2 in AC020978 downregulation cells behaved shorter half-life, while the half-life of PKM2 was much longer in AC020978-overexpressed cells than that in controls (Figure 6F), suggesting that the regulation of PKM2 by AC020978 might through inhibiting proteasome degradation.

As more than 80% of protein degradation was related to the ubiquitin-proteasome pathway 25, we tested whether AC020978 was involved in the ubiquitin-mediated PKM2 degradation by *in vitro* ubiquitination assay. Downregulation of AC020978 increased the level of ubiquitination of PKM2 protein in A549 cell. The ubiquitin-mediated degradation effect of PKM2 was significantly abrogated in AC020978 overexpression H1299 cell (Figure 6G). These collecting results demonstrated that AC020978 contributed to maintain PKM2 protein stability by ubiquitin-mediated proteasome degradation.

To further validate the role of PKM2 in AC020978's biological function, a complementary rescue experiment was performed. PKM2 plasmid was transfected into AC020978-downregulated A549 and H1299 cells, and the decrease in cell proliferation (Figure S5A-S5B), ^18^F-FDG uptake (Figure S5D-S5E) and lactate production (Figure S5G-S5H) were significantly rescued. For further confirm, PKM2 siRNA was transfected into AC020978-overexpressed H1299 cell, and AC020978-induced increase in cell proliferation (Figure S5C), ^18^F-FDG uptake (Figure S5F) and lactate production (Figure S5I) was consequently reduced. These rescue experiments indicated that the regulation of cell proliferation and glycolytic metabolism of AC020978 was depended on PKM2.

### AC020978 promotes the nuclear translocation of PKM2 and regulates PKM2-enhanced HIF-1α transactivation activity

By immunofluorescence staining, we visualized both total and nuclear PKM2 could be regulated by AC020978. In A549 negative control cells, PKM2 was predominantly present in the cytoplasm, a small quantity of PKM2 signal resided in the nucleus. While in AC020978 knockdown cells, barely any detectable nuclear PKM2 was seen. When comparing the cellular distribution PKM2 in AC020978 overexpression cells, an increased level of nuclear PKM2 could be seen (Figure 6H). Western blotting analysis of nuclear and cytosolic fractions from A549 and H1299 cells revealed that PKM2 primarily expressed in the cytosolic fractions. After silencing of AC020978 in A549 cells, there were decreased signals in nuclear and cytosolic fractions of PKM2. However, ectopic expression of AC020978 performed a certain increase level of the nuclear and cytosolic PKM2 signals, in accordance with the immunofluorescence findings (Figure 6I).

**Figure 7:**
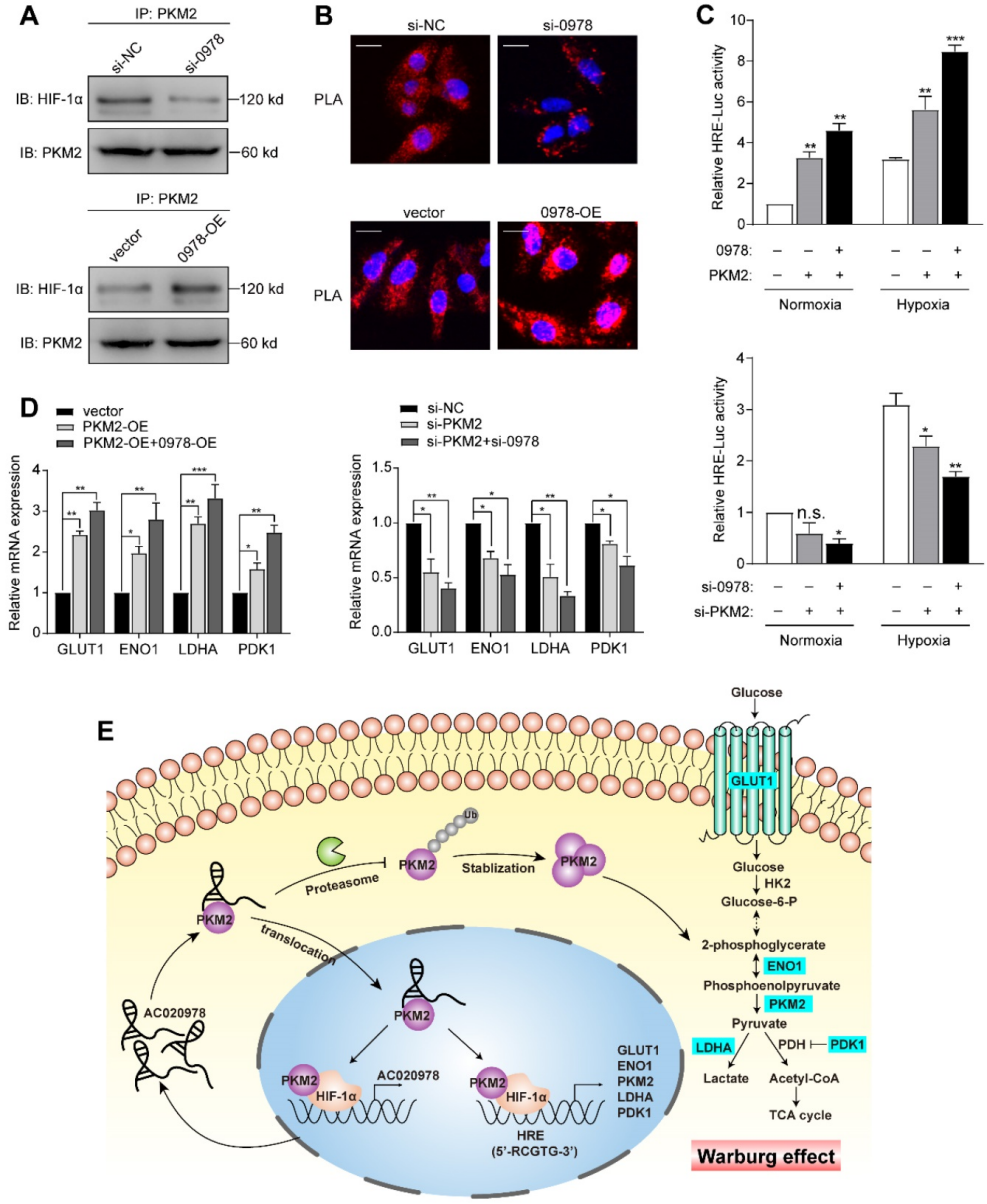
** AC020978 regulates PKM2-enhanced HIF-1α transactivation activity. (A)** Co-IP assay was performed to examine the PKM2-HIF-1α interaction in the AC020978-knockdown, AC020978-overexpressing and control groups. **(B)** *In situ* proximity ligation assay (PLA) on A549 cells demonstrated the interaction between PKM2 and HIF-1α in the AC020978-knockdown, AC020978-overexpressing and control groups. Positive PLA signals demonstrated PKM2/HIF-1α complex which were shown as red clusters, and cell nuclei were counterstained with blue (Scale bars=10 µm). **(C)** Transactivation activity of HEK293 cells co-transfected with expression vectors as indicated was measured by dual-luciferase report assay. The ratio of activity was normalized to the blank group at normoxia. **(D)** QRT-PCR analysis of GLUT1, ENO1, LDHA and PDK1 mRNAs in H1299 cells transduced with indicated plasmids or si-RNAs and exposed to 1% O2 for 24 h. **(E)** Schematic illustration of AC020978/ PKM2/ HIF-1α positive feedback loop in promoting glycolytic metabolism and tumorigenesis. Data shown are mean±SD (n = 3). (*P < 0.05, **P < 0.01, ***P < 0.001).

As a pivotal regulator of tumor metabolism, both cytosolic and nuclear PKM2 contribute to altered metabolism and proliferation in cancer. Previous studies indicated that the oncogenic role of PKM2 partly contributed to its translocation into the nucleus and act as a co-activator of HIF-1α 26. In our study, PKM2 served as the functional downstream of AC020978 and contributed to perform malignant biological behaviors, which prompted us to suspect whether AC020978 played a role in stimulating the PKM2/HIF-1α pathway. Firstly, we performed co-IP to explore whether AC020978 could regulate the interaction between PKM2 and HIF-1α. Intriguingly, knockdown AC020978 greatly impaired but AC020978 overexpression noticeably strengthened the PKM2 and HIF-1α interaction (Figure 7A). Furthermore, to visualize native protein complexes, we used the *in situ* proximity ligation assay (Figure 7B, Figure S6A). Under the condition of AC020978 silencing, less PLA-positive protein complexes that included PKM2 and HIF-1α was observed. On the contrary, an overall high density of PKM2/HIF-1α clusters was found in the AC020978 overexpression group, which is in consistent with the protein interaction results of co-IP. However, neither the RNA pull-down or RIP assay showed direct interaction between AC020978 and HIF-1α (Figure S6B-S6C).

Next, we determined whether AC020978 regulates PKM2-stimulated HIF-1α transactivity. Luciferase assay showed that ectopic expression of PKM2 could increase the HRE reporter activity either under normoxia or hypoxia, whereas co-expression of AC020978 and PKM2 synergistically enhanced the promoter activity. In a reciprocal experiment, depletion of both AC020978 and PKM2 led to a significant reduction in HIF-1α transactivity either under normoxia or hypoxia (Figure 7C). The expression of HIF-1α targeted genes involved in Warburg effect, including GLUT1, LDHA, ENO1 and PDK1 were then tested under hypoxia. The co-transfection of AC020978 and PKM2 exhibited a relatively higher expression for all of these genes; whilst the combined knockdown of AC020978 and PKM2 showed the opposite results (Figure 7D). Taken together, these data suggested that AC020978 augmented PKM2-enhanced HIF-1α transcriptional activity.

## Discussion

Numerous dysregulated genes have been widely investigated in the pathogenesis of human lung cancer, among which lncRNAs have gained widespread attention by presenting new perspectives for exploring molecular pathways in NSCLC progression 27,28. Since aberrant metabolism has been recognized as an emerging hallmark of cancer, exploring metabolic-related lncRNAs may improve understanding of the regulatory mechanisms in biological processes of NSCLC 13,29. In our previous study, RNA-seq analysis of high ^18^F-FDG uptake NSCLC tissues was performed to identify glycolysis associated lncRNAs 18. Among the top-scoring differentially upregulated lncRNAs, AC020978, is a newly identified oncogenic lncRNA and significantly associated with poor clinical outcomes of NSCLC patients. Clinical analysis pointed to the notion that AC020978 might represent as an independent prognostic biomarker of NSCLC.

As most cancer cells are sensitive to glucose deprivation and hypoxic conditions, one would wonder how cancer cells adapt to survive under the cellular stress microenvironment. Previous researches have indicated that metabolic plasticity is essential for cancer cells to survive hostile nutrient deprived environments. Cancer cells might select for highly metabolically plastic cells to survive 30,31. Our current study demonstrated that AC020978 was a stress-responsive gene and presented as a potential key to coordinate metabolic reprogramming. These results indicated that AC020978 facilitating metabolic plasticity and conferring a proliferation advantage under metabolic stress. Moreover, the expression of AC020978 was also raised under hypoxia. Mechanistic assay illustrated that it was a direct transcriptional target of HIF-1α. The critical involvement of lncRNA in hypoxia-associated energy metabolism has been vigorously studied recently 16,17. For instance, lincRNA-p21 was reported as a hypoxia-responsive lncRNA which could in turn regulate HIF-1α protein stability. This positive feedback loop reciprocally promoted glycolysis under hypoxia 32. Another study showed that LncHIFCAR, a hypoxia-inducible lncRNA, formed a complex with HIF-1α via direct binding and facilitated the recruitment of HIF-1α and p300 cofactor to the target promoters, thus was crucial for metabolic shifting and progression of oral carcinoma 22. As a consequence, we inferred that AC020978 was a potential key lesion to coordinate the metabolic reprogramming under glucose stress and hypoxic conditions.

Accumulating evidence has illustrated that lncRNAs participate in global cellular behaviors by binding with specific proteins 33. Regulation roles of lncRNAs in the lncRNA-protein interaction involve various aspects, such as modulating protein expression and activity, altering protein localization or serving as structural components 34. When we explored the protein interacted with AC020978 by RNA-pulldown assay, PKM2 drew our interest. Located at a “gate” position in the glycolytic flux, PKM2 is crucial for the Warburg effect. Abnormal expression and localization of PKM2 have been described in numerous kinds of human cancers 19. Recent studies have reported that PKM2 could function as pyruvate enzyme or protein kinase to promote tumorigenesis and progression by interacting with lncRNAs in several tumor types. Bian et al. 35 concluded that LncRNA-FEZF1-AS1 could increase both total and nuclear PKM2 activity and consequently activate STAT3 to perform tumor-promoting role in colorectal cancer. Another research suggested that LINC01554 suppressed glucose metabolism reprogramming via downregulating PKM2 expression in hepatocellular carcinoma 36. Consistent with the previous studies, our study confirmed that PKM2 was functionally responsible for AC020978-mediated NSCLC progression. In return, AC020978 served to stabilize PKM2 protein through ubiquitin mediated degradation.

In addition to its central role as a pyruvate enzyme at cytoplasm, PKM2 may also translocate into the nucleus to function as a protein kinase and interact with many tumor-associated genes to accelerate carcinogenesis, including HIF-1α, Oct-4, STAT3 and beta-catenin 26,37-39. We observed that AC020978 could increase both total and nuclear PKM2, and enhance the PKM2/HIF-1α interaction, consequently modulate gene transcription in the HIF-1α system. According to previous reviews, lncRNAs have emerged as key components of the address code, allowing proteins, genes, and chromosomes to be trafficked to appropriate locations and subject to proper activation and deactivation 24. Several characteristics of lncRNAs make them the ideal system to provide molecular addresses to the nucleus. On the one hand, a RNA-based address code may be deployed more rapidly and economically than a system that relies on proteins. On the other hand, the capacity of lncRNAs to function as protein coupling endows them with enormous regulatory potential in gene expression and for spatial control within the cell 40. In this study, AC020978 was speculated to serve as an address code to direct PKM2 to translocate into the nucleus. In the oxygen-glucose deprivation microenvironment, HIF-1α is a master regulator that plays crucial roles for tumor survival. Correlation between PKM2 and HIF-1α is complicated. According to Wang's study 41, JMJD5 took part in regulating PKM2 nuclear translocation and reprogramming HIF-1α-mediated glucose metabolism. Luo et al. 26 reported that the hydroxylation of PKM2 by PHD3 stimulated its nuclear translocation, as well as enhanced its direct binding with HIF-1α, thus promoting the transcription of HIF-1α targeted genes. Briefly, our data uncovered the diverse and complex functions of lncRNA in modulating the PKM2/HIF-1α complex and further promoting HIF-1α transactivating targeted genes *in vitro*. Since no direct interaction was observed between AC020978 and HIF-1α, it is speculated that AC020978 might act as a molecular chaperone for PKM2 to interact with HIF-1α.

The hypoxia induced up-regulation of AC020978, coupled with the synergistically AC020978-PKM2 stimulated HIF-1α transcription activity, might result in a positive feedback loop (Figure 7E). The aberrant activation of AC020978/PKM2/HIF-1α glycolysis cascade might be cancer's comprehensive metabolic adaptation to allow cell survival under glucose starvation and hypoxia cellular stress. In our next strategy, additional efforts should be made to explore how AC020978 regulates the sub-cellular location of PKM2, and the reconstitution of glycolysis pathways in NSCLC cells orchestrated by AC020978.

## Conclusions

Taken together, AC020978 is an upregulated lncRNA of clinical significance in NSCLC, which holds potential to be an independent biomarker of prognostic prediction in NSCLC. As a direct transcriptional target of HIF-1α, it functions as an oncogene in promoting proliferation as well as glycolysis pathway under glucose starvation and hypoxic conditions. In this study, we identify that AC020978 directly binds with PKM2 and stabilizes PKM2 protein through ubiquitin mediated degradation. Furthermore, it was capable of modulating the interaction of PKM2/HIF-1α complex, and further enhancing the transactivity of HIF-1α to its target genes. The positive feedback loop of AC020978/PKM2/HIFα axis paves the way to cancer-specific metabolism in response to low oxygen-glucose challenge. Therefore, from a therapeutic perspective, targeting this AC020978/PKM2/HIF-1α axis may present new perspectives in the prevention or treatment of NSCLC.

## Supplementary Material

Supplementary materials and methods, figures, and tables.

## Abbreviations

lncRNA: long noncoding RNA
NSCLC: non-small cell lung cancer
FDG: fluorodeoxyglucose
OS: overall survival
TNM: tumor-node-metastasis staging system
CI: confidence interval
HR: hazard ratio
ROC: receiver operating characteristic curve
AUC: area under curve
RIP: RNA immunoprecipitation
ChIP: Chromatin immunoprecipitation
FISH: fluorescence *in situ* hybridization
IHC: immunohistochemistry
IF: Immunofluorescence
PLA: Proximity Ligation Assay
ECAR: extracellular Acidification Rate
OCR: Oxygen Consumption Rate
PKM2: Pyruvate kinase isozymes M2
GLUT1: glucose transporter-1
LDHA: lactate dehydrogenase A
PDK1: pyruvate dehydrogenase kinase 1
ENO1: enolase 1
HK2: hexokinase II
HIF-1: hypoxia-inducible factor-1
OXPHOS: mitochondrial oxidative phosphorylation
