# Supplementary material for: Hypoxia-induced lncRNA-AC020978 promotes proliferation and glycolytic metabolism of non-small cell lung cancer by regulating PKM2/HIF-1α axis
Source: Theranostics. 2020 Mar 26;10(11):4762–78. doi: 10.7150/thno.43839 (PMC7163453; doi:10.7150/thno.43839)
Supplement: Supplementary file 1 — Supplementary materials and methods, figures, and tables. [file thnov10p4762s1.pdf]

Supplementary Materials and Methods

5'and 3' Rapid Amplification of cDNA Ends (RACE) analysis

5'RACE and 3'RACE were performed using SMARTer™ RACE cDNA kit (Clontech) according to the manufacturer's instructions. Briefly, two sets of primers were designed and synthesized for the nested PCR. The RACE PCR products were separated on a 1.5% agarose gel. The results of electrophoresis were confirmed, and the amplified bands were sequenced bi-directionally using the indicated primers. The gene-specific primers (GSP) used for PCR are presented in Table S1.

Colony formation assay

As for the colony formation assay, a total of 1000 cells were seeded in 6-well plates and cultured in a humidified atmosphere containing 5% CO_2 at 37 ℃ for 2 weeks. Cell colonies were washed with PBS, fixed with 4% paraformaldehyde, and stained with 0.1% crystal violet (1 mg/mL) for 20 min. All the experiments were repeated in triplicate and the mean was calculated.

Transwell assay

Transwell assay was performed using Boyden chambers containing a transwell membrane filter (Corning). Briefly, 1×10^5 cells were suspended in 200 μl of Dulbecco's modified Eagle's medium with 1% fetal bovine serum and then put into the upper chamber per well. The lower chamber was completed by 600 μl of Dulbecco's modified Eagle's medium with 10% fetal bovine serum. After incubation at 37 ℃ and 5% CO_2 for 48 h, cells that moved to the bottom surface of the upper chamber were fixed with 4% formalin for 20 min and stained with 0.1% crystal violet for 30 min. At least five random fields of view were analyzed for each chamber. The results were photographed using an inverted microscope (Olympus) and analyzed by

ImageJ software.

Supplementary Figures:

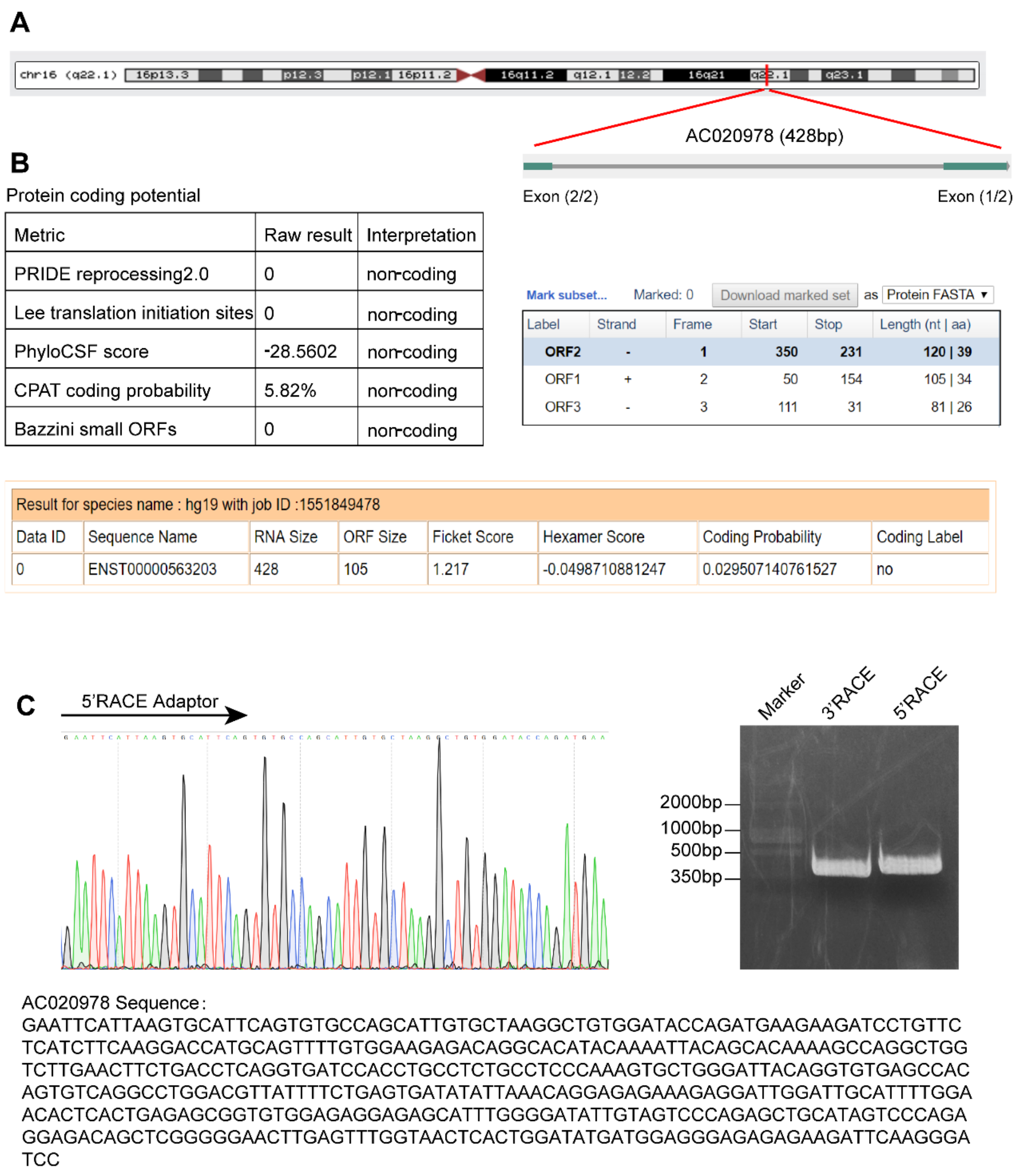

Figure S1. The non-coding nature and whole sequence of AC020978.

(A) AC020978 is located on chromosome 16 in humans and composed of two exons with a full length of 428 bp. (B) The coding potential of AC020978 in several prediction softwares, and results showed that AC020978 didn't have any coding potential. (C) 5' and 3' rapid

amplification of cDNA ends (RACE) assays in A549 cells to detect the whole sequence of AC020978. The sequencing of 5'-RACE PCR products reveals the boundary between the universal anchor primer and the AC020978 sequences (left). The red arrow indicates a putative transcriptional start site. Agarose gel electrophoresis of PCR products from the 5'-RACE and 3'-RACE procedure (right). The AC020978 sequence is shown at the bottom.

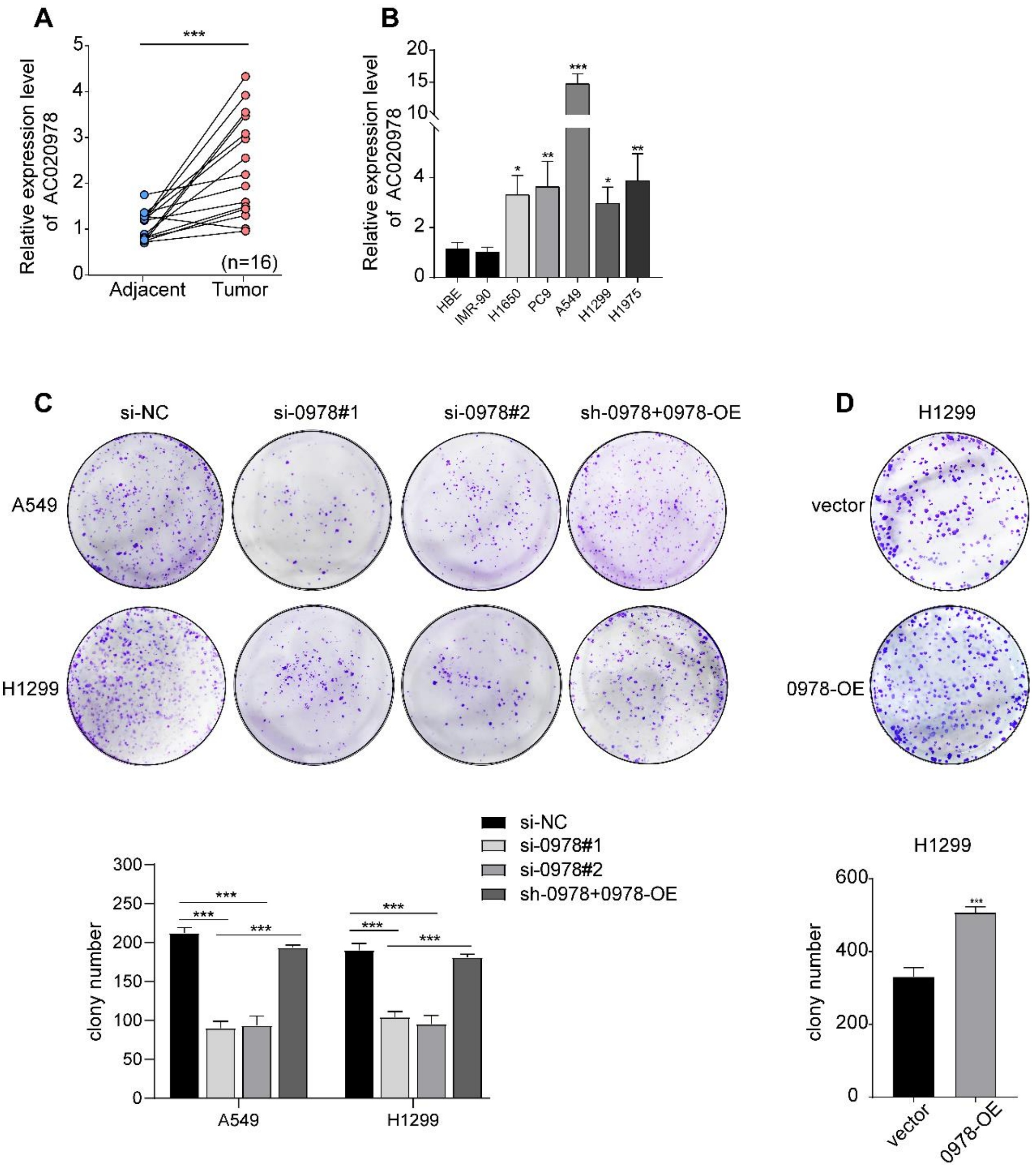

Figure S2. The expression pattern of AC020978 and its oncogenic roles in promoting cell

proliferation of NSCLC cells.

(A) Expression of AC020978 in 16 paired NSCLC tissues and adjacent normal tissues was analyzed by qRT-PCR. (B) Expression of AC020978 in five NSCLC cell lines (A549, H1299, H1650, H1975, PC9) and two normal lung cell line (HBE and IMR-90) was analyzed by qRT-PCR. (C) Cell proliferation was performed by colony formation assay in A549 and H1299 cells transfected with negative control group (si-NC), AC020978 siRNAs group (si-0978#1, si-0978#2) and rescue group (sh-0978+0978-OE). (D) Cell proliferation was performed by colony formation assay in H1299 cells transfected with pcDNA-0978 or vector control. Data shown are mean ±SD (n = 3). (*P < 0.05, **P < 0.01, ***P < 0.001)

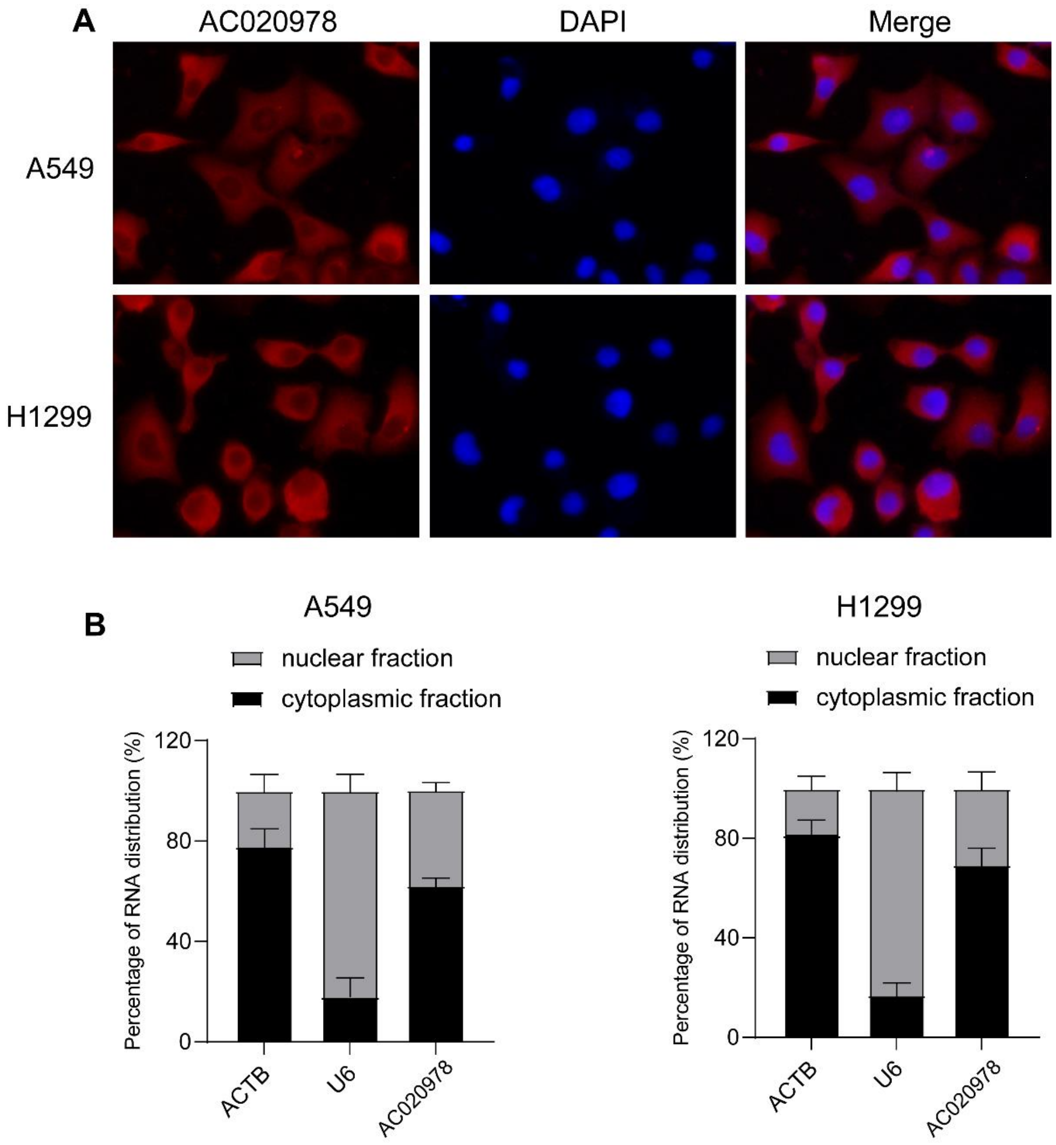

Figure S3. AC020978 mainly locates at cytoplasm.

(A) Representive FISH images showed the sub-cellular distribution of AC020978 in A549 and H1299 cells (red). Nuclei were stained by DAPI (blue). (B) Relative AC020978 expression levels in nuclear and cytosolic fractions of A549 and H1299 cells was analyzed by qRT-PCR. Nuclear controls: U6, cytosolic controls: ACTB. Data shown are mean ±SD (n = 3).

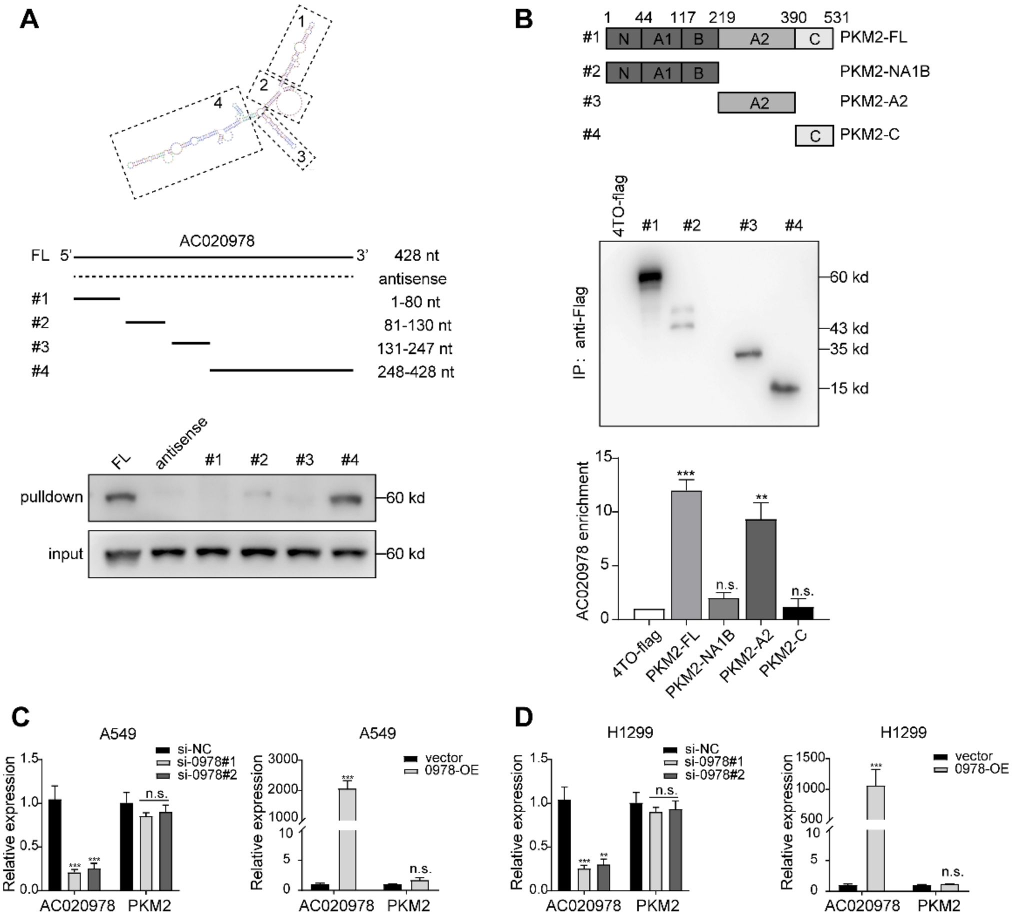

Figure S4. AC020978 directly binds with PKM2 protein.

(A) Immunoblot detection of the PKM2 protein in A549 cells as retrieved by in vitro transcribed biotinylated RNAs of different constructs of AC020978 or its antisense sequence (negative control). (B) RIP assay was performed using an anti-Flag antibody in 293T cells transfected with Flag-tagged PKM2 vector or its deletion mutants (4TO-Flag vector as negative control). QRT-PCR was used to measure the enrichment of AC020978. Western blot was used to evaluate the expression of Flag-tagged PKM2 or its deletion mutants. (C) PKM2 expression level was measured by qRT-PCR after knockdown or overexpression of AC020978 in A549 cell. (D) PKM2 expression level was measured by qRT-PCR after knockdown or overexpression of AC020978 in H1299 cell. Data shown are mean±SD (n = 3). (*P < 0.05, **P < 0.01, ***P < 0.001)

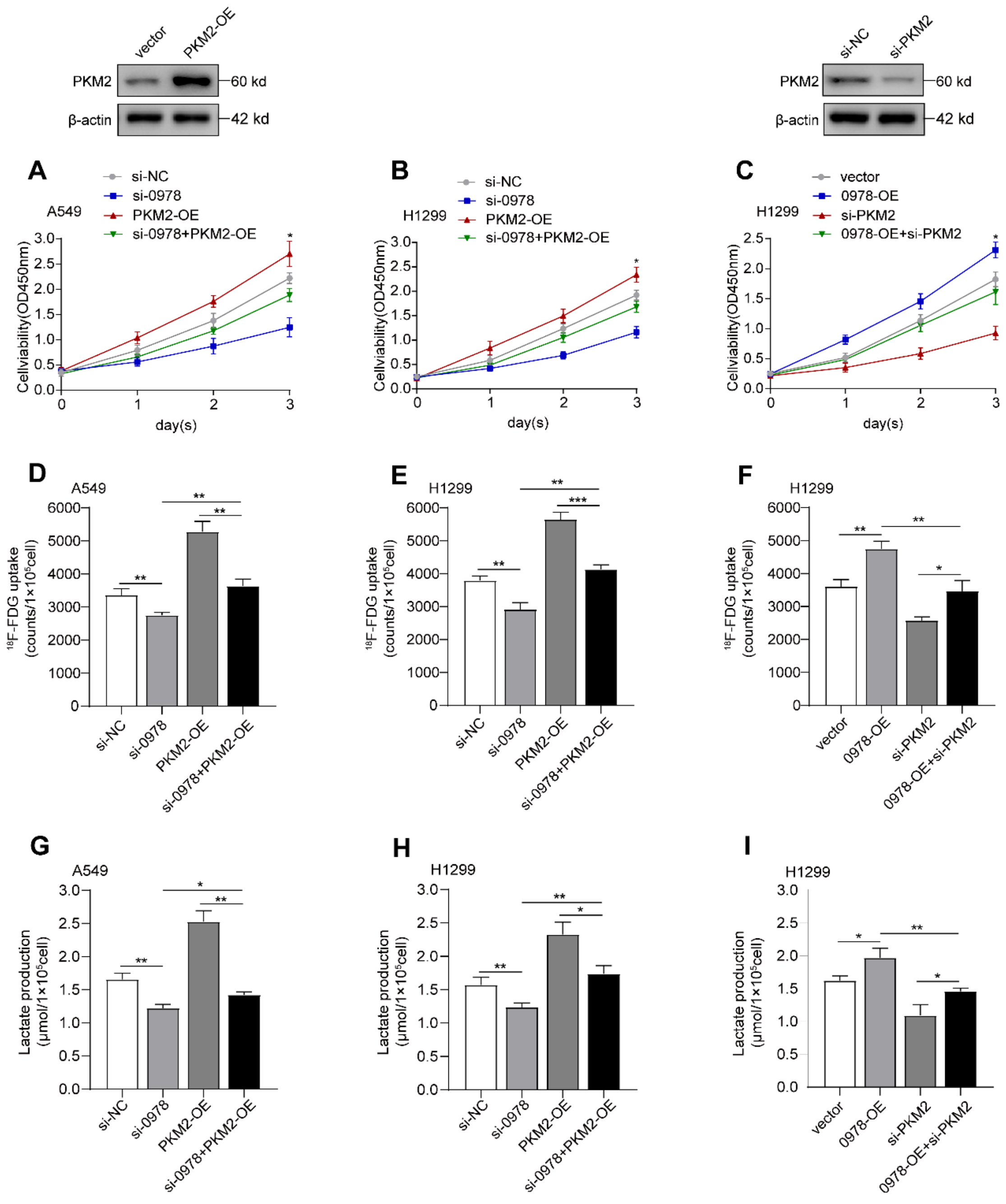

Figure S5. PKM2 participates in the biological function of AC020978 in NSCLC cells.

(A-B) Cell proliferation was performed in A549 (A) and H1299 (B) cells co-transfected with control or si-0978 with PKM2 overexpression plasmid by CCK-8 assay. (C) Cell proliferation was performed in H1299 cell co-transfected with vector control or pcDNA-0978 with si-PKM2 by CCK-8 assay. (D-E) ^{18}F-FDG uptake level was determined in A549 (D) and H1299 (E) cells co-transfected with control or si-0978 with PKM2 overexpression plasmid. (F) ^{18}F-FDG uptake

level was determined in H1299 cell co-transfected with vector control or pcDNA-0978 with si-PKM2. (G-H) Lactate release level was determined in A549 (G) and H1299 (H) cells co-transfected with control or si-0978 with PKM2 overexpression plasmid. (I) Lactate release level was determined in H1299 cell co-transfected with vector control or pcDNA-0978 with si-PKM2. Data shown are mean ±SD (n = 3). (*P < 0.05, **P < 0.01, ***P < 0.001)

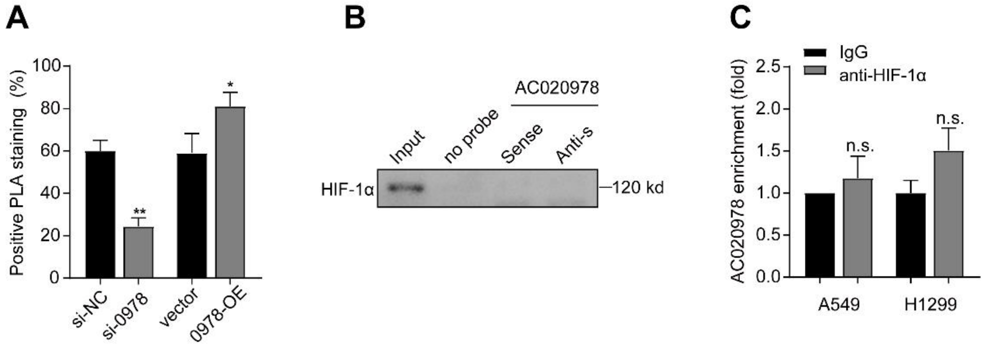

Figure S6. AC020978 does not directly bind with HIF-1α.

(A) Quantification of the positive PLA staining between si-NC and si-0978 groups, as well as between vector and 0978-OE group. (B) Western blot analysis of the proteins retrieved from the AC020978 pull-down assay using an anti-HIF-1α antibody. (C) RIP assay using an anti-HIF-1α antibody showed that HIF-1α did not interact with AC020978 in A549 cells. Data shown are mean ±SD (n = 3). (*P < 0.05, **P < 0.01, ***P < 0.001)

Supplementary Tables:

Table S1. Primers used in the paper were listed:

Gene	Primer	Sequence(5′-3′)
**Primers for qRT-PCR**		
ACTB	forward	CATGTACGTTGCTATCCAGGC
	reverse	CTCCTTAATGTCACGCACGAT
AC020978	forward	GGACCATGCAGTTTTGTGGAAGA
	reverse	GGCAGAGGCAGGTGGATCAC
GLUT1	forward	ATGAACTACCCTCACTCCAGC
	reverse	TATTGGACACAGCTTGGATGCC
LDHA	forward	AGGAGAAACACGCCTTGATTTAG
	reverse	ACGAGCAGAGTCCAGATTACAA
PKM2	forward	GGGCCATAATCGTCCTCACC
	reverse	TTGCACAGCACAGGGAAGAT
PDK1	forward	GGTGTTTACCCCCCTATTCAAG
	reverse	CGGGAGGTCTCAACACGA
ENO1	forward	AAAGCTGGTGCCGTTGAGAA
	reverse	GGTTGTGGTAAACCTCTGCTC
U6	forward	CTCGCTTCGGCAGCACA
	reverse	CTCAACTGGTGTCGTGGA
**Sequences for 5' and 3'RACE**		
3'-RACE GSP1	5' CTGGGATTACAGGTGTGAGCCACAGTG 3'	
3'-RACE GSP2	5' GGCTGTGGATACCAGATGAAGAAGATCC 3'	
5'-RACE GSP1	5' TGCTCTCCTCTCCACACCGCTCTC 3'	
5'-RACE GSP2	5' CCTCTGGGACTATGCAGCTCTGGG 3'	
**Sequences for gene knockdown**		
si-0978#1	forward	CCAGCAUUGUGCUAAGGCUTT
	reverse	AGCCUUAGCACAAUGCUGGTT
si-0978#2	forward	GAGAGAAAGAGGAUUGGAUTT

	reverse	AUCCAAUCCUCUUUCUCUCTT
si-HIF-1α#1	forward	GUGAUGAAAGAAUUACCGAAUTT
	reverse	AUUCGGUAAUUCUUUCAUCACTT
si-HIF-1α#2	forward	CCUAUAUCCCAAUGGAUGAUGTT
	reverse	CAUCAUCCAUUGGGAUAUAGGTT
si-PKM2	forward	GCCCGAGGCUUCUUCAAGAAGTT
	reverse	CUUCUUGAAGAAGCCUCGGGCTT
sh-0978	CCGGCCAGCATTGTGCTAAGGCTTTCTCGAGAGCCTTAG CACAATGCTGGTTTTTTTG	

Primers for ChIP-qPCR

ChIP-0978 promoter 1-1	forward	CGCACAATCATGGCTCACTG
	reverse	GGCACAGTAGCTGACGCCTAA
ChIP-0978 promoter 1-2	forward	TCAAGTGATCCTCCCGCCT
	reverse	TTGAGGCTGGGCACAGTAG
ChIP-0978 promoter 2-1	forward	TTAGGTCCTCACTGCTGTATCC
	reverse	GGCGTGACGGCCTCATTT
ChIP-0978 promoter 2-2	forward	TACCTGGCATATCGTAGGCAC
	reverse	CCCAGTCGCAGCACAGTTTC

Sequences of probes for AC020978 RNA FISH

5’-DIG-TCTTCTTCATCTGGTATCCACAGCCTTAGCACAAT-DIG-3’

Table S2. Primary antibodies used in the paper were listed:

Name	Company	Cat. No.	Concentration
Anti-PKM2 antibody	CST Biologicals	#3198	1:1000 (WB)
Anti-PKM2 antibody	Proteintech	60268-1-lg	1:200 (IF)
Anti-PKM2 antibody	Proteintech	60268-1-lg	1:500 (IHC)
Anti-PKM2 antibody	Proteintech	60268-1-lg	2 μg (IP)
Anti-HIF1A antibody	Proteintech	20960-1-AP	1:500 (WB)
Anti-HIF1A antibody	Proteintech	20960-1-AP	1:500 (IHC)
Anti-HIF1A antibody	Proteintech	20960-1-AP	4 μg (ChIP)
Anti-GLUT1 antibody	Proteintech	21829-1-AP	1:1000 (WB)
Anti-HK2 antibody	Proteintech	22029-1-AP	1:1000 (WB)
Anti-LDHA antibody	Proteintech	19987-1-AP	1:4000 (WB)
Anti-PDK1 antibody	Proteintech	10026-1-AP	1:2000 (WB)
Anti-ENO1 antibody	Proteintech	11204-1-AP	1:1000 (WB)
Anti-flag antibody	Proteintech	20543-1-AP	1:1000 (WB)
Anti-HA antibody	Biolegend	901503	1:2000 (WB)
Anti-ACTB antibody	Proteintech	60008-1-lg	1:10000 (WB)
Anti-Lamin B1 antibody	CST Biologicals	#13435	1:1000 (WB)
Anti-Alpha Tubulin antibody	Proteintech	66031-1-lg	1:2000 (WB)

Table S3. Correlation of the expression of AC020978 in NSCLC with clinicopathologic features

Characteristics		AC020978		P value
		Low (n)	High (n)	
Age (years)				
<55	32 (34.8%)	13	19	0.55
≥55	60 (65.2%)	25	35	
Gender				
Male	51 (55.4%)	20	31	0.40
Female	41 (44.6%)	18	23	
TNM stage				
I	49 (53.3%)	27	22	<0.01**
II	19 (20.7%)	7	12	
III	22 (23.9%)	4	18	
IV	2 (2.2%)	0	2	
T stage				
T1	37 (40.2%)	25	12	<0.01**
T2	37 (40.2%)	12	25	
T3	11 (12.0%)	1	10	
T4	7 (7.6%)	0	7	
Lymph node metastasis				
N0	55 (59.8%)	31	24	<0.01**
N1	22 (23.9%)	5	17	
N2	11 (12.0%)	2	9	
N3	4 (4.3%)	0	4	
Distant metastasis				
M0	90 (97.8%)	38	52	0.34
M1	2 (2.2%)	0	2	
Mortality				
Survive	41 (44.6%)	24	17	<0.01**
die	51 (55.4%)	14	37	
histological grade				
I	2 (2.2%)	0	2	0.33
II	57 (62.0%)	27	30	
III	33 (35.9%)	11	22	
Tumor location				
left	35 (38.0%)	16	19	0.32
right	57 (62.0%)	22	35	

*P<0.05; **P<0.01 (AC020978 high expression: score 7-12; low expression: score 0-6)

Table S4. Univariate and multivariate Cox regression analysis for clinicopathological features association with prognosis of 92 NSCLC patients

Variables	Univariate analysis		Multivariate analysis	
	HR (95%CI)	P	HR (95%CI)	P
Age (≥55 vs. < 55)	1.294 (0.715-2.339)	0.394	1.859 (0.923-3.744)	0.083
Gender (Male vs. Female)	1.445 (0.823-2.537)	0.200	1.399 (0.771-2.539)	0.270
TNM stage (I vs. II-IV)	3.045 (1.720-5.391)	0.001**	3.795 (1.659-8.685)	0.002**
T stage (T1 vs. T2-4)	2.300 (1.233-4.290)	0.009**	0.728 (0.301-1.759)	0.480
Lymph node metastasis (N0 vs. N1-3)	1.823 (1.051-3.163)	0.033*	0.697 (0.302-1.611)	0.399
Distant metastasis (M0 vs. M1)	0.393 (0.094-1.641)	0.200	1.289 (0.274-6.064)	0.748
Histologic grade (I vs. II-III)	1.373 (0.782-2.411)	0.269	1.478 (0.800-2.734)	0.212
Tumor location (Left vs. Right)	0.936 (0.530-1.654)	0.820	0.949 (0.522-1.728)	0.865
AC020978 (low vs. high)	2.503 (1.349-4.643)	0.004**	2.274 (1.184-4.366)	0.014*

*P<0.05; **P<0.01

Table S5. Mass spectrometry analysis for RNA pull-down.

**Accession**	**Description**	**Score**	**Coverage**	**MW [kDa]**	**calc. pI**
P10809	60 kDa heat shock protein, mitochondrial OS=Homo sapiens GN=HSPD1 PE=1 SV=2 - [CH60_HUMAN]	352.496307	23.91	61.01638505	5.871582031
P04083	Annexin A1 OS=Homo sapiens GN=ANXA1 PE=1 SV=2 - [ANXA1_HUMAN]	284.8787859	18.79	38.68998096	7.020019531
P0DMV8	Heat shock 70 kDa protein 1A OS=Homo sapiens GN=HSPA1A PE=1 SV=1 - [HS71A_HUMAN]	157.8689827	6.24	70.00904046	5.655761719
P14618	Pyruvate kinase PKM OS=Homo sapiens GN=PKM PE=1 SV=4 - [KPYM_HUMAN]	136.8328746	14.07	57.90002631	7.840332031
P40926	Malate dehydrogenase, mitochondrial OS=Homo sapiens GN=MDH2 PE=1 SV=3 - [MDHM_HUMAN]	123.0258729	9.76	35.48073441	8.675292969
Q15084	Protein disulfide-isomerase A6 OS=Homo sapiens GN=PDIA6 PE=1 SV=1 - [PDIA6_HUMAN]	92.05643617	9.09	48.09126437	5.084472656
P00352	Retinal dehydrogenase 1 OS=Homo sapiens GN=ALDH1A1 PE=1 SV=2 - [AL1A1_HUMAN]	72.18327059	11.18	54.82695038	6.727050781
P32119	Peroxiredoxin-2 OS=Homo sapiens GN=PRDX2 PE=1 SV=5 -	50.18205834	5.56	21.87823885	5.973144531

	[PRDX2_HUMAN]				
Q9NQC3	Reticulon-4 OS=Homo sapiens GN=RTN4 PE=1 SV=2 - [RTN4_HUMAN]	47.23	1.09	129.8511941	4.500488281
P09525	Annexin A4 OS=Homo sapiens GN=ANXA4 PE=1 SV=4 - [ANXA4_HUMAN]	46.19491722	11.29	35.86012254	6.125488281
P14625	Endoplasmin OS=Homo sapiens GN=HSP90B1 PE=1 SV=1 - [ENPL_HUMAN]	39.8784476	4.61	92.41134842	4.843261719
P05089	Arginase-1 OS=Homo sapiens GN=ARG1 PE=1 SV=2 - [ARGI1_HUMAN]	39.84	3.42	34.7132988	7.210449219
P23528	Cofilin-1 OS=Homo sapiens GN=CFL1 PE=1 SV=3 - [COF1_HUMAN]	39.32	7.23	18.49065867	8.089355469
P68104	Elongation factor 1-alpha 1 OS=Homo sapiens GN=EEF1A1 PE=1 SV=1 - [EF1A1_HUMAN]	37.92	2.38	50.10911075	9.012207031
P27824	Calnexin OS=Homo sapiens GN=CANX PE=1 SV=2 - [CALX_HUMAN]	36.15	2.7	67.52585354	4.602050781
P61626	Lysozyme C OS=Homo sapiens GN=LYZ PE=1 SV=1 - [LYSC_HUMAN]	34.26	8.11	16.52628464	9.158691406
Q08554	Desmocollin-1 OS=Homo sapiens GN=DSC1 PE=1 SV=2 - [DSC1_HUMAN]	31.1	1.68	99.92375345	5.427246094
P06748	Nucleophosmin OS=Homo sapiens GN=NPM1 PE=1 SV=2 - [NPM_HUMAN]	30.14	3.06	32.55484332	4.779785156
P11021	78 kDa glucose-regulated protein OS=Homo sapiens GN=HSPA5 PE=1 SV=2 - [GRP78_HUMAN]	29.62	1.51	72.28843987	5.160644531
P06733	Alpha-enolase OS=Homo sapiens GN=ENO1 PE=1 SV=2 - [ENOA_HUMAN]	29.46	6.91	47.13932161	7.386230469
P23284	Peptidyl-prolyl cis-trans isomerase B OS=Homo sapiens GN=PPIB PE=1 SV=2 - [PPIB_HUMAN]	29.33	6.02	23.72753677	9.407714844
P63104	14-3-3 protein zeta/delta OS=Homo sapiens GN=YWHAZ PE=1 SV=1 - [1433Z_HUMAN]	28.16	4.9	27.72772965	4.792480469
P25705	ATP synthase subunit alpha, mitochondrial OS=Homo sapiens GN=ATP5A1 PE=1 SV=1 -	26.5	4.16	59.71359642	9.129394531

	[ATPA_HUMAN]				
P08238	Heat shock protein HSP 90-beta OS=Homo sapiens GN=HSP90AB1 PE=1 SV=4 - [HS90B_HUMAN]	25.37	3.59	83.21210592	5.033691406
P13804	Electron transfer flavoprotein subunit alpha, mitochondrial OS=Homo sapiens GN=ETFA PE=1 SV=1 - [ETFA_HUMAN]	24.49	3.9	35.05758373	8.382324219
Q96EZ8	Microspherule protein 1 OS=Homo sapiens GN=MCRS1 PE=1 SV=1 - [MCRS1_HUMAN]	22.55	2.81	51.77120586	9.378417969
P61604	10 kDa heat shock protein, mitochondrial OS=Homo sapiens GN=HSPE1 PE=1 SV=2 - [CH10_HUMAN]	21.87	13.73	10.92487173	8.924316406
Q08211	ATP-dependent RNA helicase A OS=Homo sapiens GN=DHX9 PE=1 SV=4 - [DHX9_HUMAN]	0	1.42	140.8691152	6.844238281
Q9Y6V0	Protein piccolo OS=Homo sapiens OX=9606 GN=PCLO PE=1 SV=5 - [PCLO_HUMAN]	0.18	1	6.468261719	
